# Imaging multicellular specimens with real-time optimized tiling light-sheet selective plane illumination microscopy

**DOI:** 10.1038/ncomms11088

**Published:** 2016-03-23

**Authors:** Qinyi Fu, Benjamin L. Martin, David Q. Matus, Liang Gao

**Affiliations:** 1 Department of Chemistry, Stony Brook University, Stony Brook, New York 11794, USA; 2 Department of Biochemistry & Cell Biology, Stony Brook University, Stony Brook, New York 11794, USA

## Abstract

Despite the progress made in selective plane illumination microscopy, high-resolution 3D live imaging of multicellular specimens remains challenging. Tiling light-sheet selective plane illumination microscopy (TLS-SPIM) with real-time light-sheet optimization was developed to respond to the challenge. It improves the 3D imaging ability of SPIM in resolving complex structures and optimizes SPIM live imaging performance by using a real-time adjustable tiling light sheet and creating a flexible compromise between spatial and temporal resolution. We demonstrate the 3D live imaging ability of TLS-SPIM by imaging cellular and subcellular behaviours in live *C. elegans* and zebrafish embryos, and show how TLS-SPIM can facilitate cell biology research in multicellular specimens by studying left-right symmetry breaking behaviour of *C. elegans* embryos.

## Abstract (web summary)

Selective plane illumination microscopy (SPIM) is capable of high-resolution, high-speed 3D imaging of single cells, but application to multicellular samples is challenging. Here the authors develop tiling light sheet SPIM to image large multicellular specimens in 3D with subcellular resolution.

## Graphical abstract

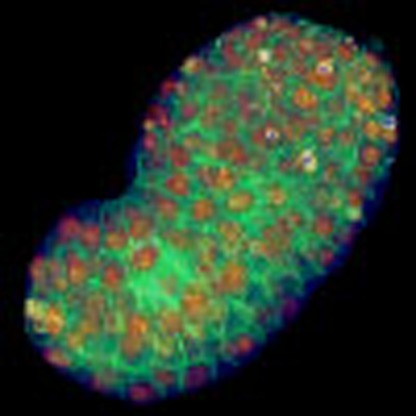

Cells live in three-dimensional (3D) environments. A more accurate understanding of cell behaviours and cell–cell interactions can be obtained by studying cells in their native, multicellular environments than when cultured on substrates. In a multicellular organism, the intracellular activities of a cell affect not only the cell itself but also its neighbour cells, and the cellular behaviour of a cell results from both its own intracellular activities and its interactions with other cells. In other words, cell behaviours in a multicellular process are caused by the intracellular activities of all cells involved in the process. Thus, in order to understand cell behaviours in a multicellular process, it is necessary to study all involved cells at subcellular level to acquire the dynamic information of their intracellular activities, by which the underlying connections between cellular behaviours and intracellular activities of the involved cells can be revealed. For this reason, fluorescence imaging techniques that allow low-invasive 3D imaging of multicellular specimens with high spatial and temporal resolution are required.

Multicellular specimens are difficult to image in 3D because of their size and complexity accompanied with optical aberrations and light scattering. Both high 3D spatial resolution and good optical sectioning capability are required in a large field of view (FOV) to visualize the specimen with subcellular, or even cellular structural details. Meanwhile, the photobleaching and photodamage must be low enough to allow live imaging at needed speed for a certain period of time. Therefore, selective plane illumination microscopy (SPIM) is getting increasing attention for its advanced 3D live imaging ability^1^,^2^,^3^,^4^. Generally, the latest SPIM techniques allow low-invasive, high-speed 3D imaging of either single-cell specimens with subcellular level, submicron 3D spatial resolution^5^,^6^,^7^,^8^ or multicellular specimens with cellular level spatial resolution of a few microns^9^,^10^,^11^,^12^, although the actual performance varies depending on the sample and fluorophore. However, imaging multicellular specimens in 3D with subcellular spatial resolution remains challenging despite the progress that has been made in SPIM development.

SPIM obtains 3D imaging ability by confining the illumination light near the detection focal plane with a light sheet. With a given detection numerical aperture (NA), its 3D imaging ability, including the spatial resolution, optical sectioning capability, FOV, photobleaching and photodamage, is mainly determined by the intensity profile of the light sheet^13^,^14^. A uniformly thin and large light sheet is therefore required in SPIM to maximize its imaging ability, and the generation of such a light sheet has been a major focus of SPIM development^5^,^6^,^7^,^15^,^16^. Although different methods have been developed, none of them are ideal. Essentially, every light sheet balances the properties of light sheet thickness, the illumination light confinement, and the light-sheet size differently, which results in different 3D spatial resolution, optical sectioning capability and FOV, respectively^13^,^14^ (Supplementary Fig. 1). Nevertheless, it becomes extremely difficult to balance these properties as the FOV increases to image multicellular specimens of dozens of microns or larger, since an ideal light sheet that has thin thickness, good light confinement and a large size at the same time does not exist due to the diffraction of light. Either the spatial resolution, optical sectioning capability, or both must be sacrificed to reach a larger FOV because the light sheet either becomes thicker, or the excitation light is less confined as its size increases. Therefore, the tradeoff between the spatial resolution, optical sectioning capability and FOV sets a fundamental limit on conventional SPIM, and a key problem of imaging multicellular specimens with subcellular spatial resolution using SPIM turns out to be how to increase the FOV without losing the spatial resolution and optical sectioning capability. Different approaches other than finding a perfect light sheet must be made.

Multiview SPIM is a different approach that works in two ways to improve the 3D imaging ability of SPIM on multicellular specimens. First, by sending the excitation light sheet and collecting the fluorescence signal from different directions, the final image is less affected by the optical aberration and light scattering introduced by the sample^9^,^10^,^11^,^12^. Next, 3D images taken from different directions can be fused together with similar methods used in tomography, by which the 3D spatial resolution can exceed the resolution limit set up by the light sheet in theory^1^,^8^,^12^. Nevertheless, the improvement in resolution by this approach relies on the number of different view directions, and the spatial resolution and the signal to noise ratio (SNR) of the 3D images taken in these different views. In multiview SPIM, the number of view directions is limited to two (lateral and axial) without rotating the sample, while the spatial resolution and SNR of the 3D image taken in each view are still strictly constrained by the light sheet used to take the image. Therefore, multiview SPIM cannot bypass the fundamental tradeoff of SPIM set up by the light sheet. Furthermore, both high-speed subcellular dynamics and the optical aberration and light scattering introduced by the sample and the agarose gel usually used to mount the sample make it more difficult to fuse different views accurately with high spatial resolution. Consequently, multiview SPIM is suited for imaging relatively large and bright multicellular specimen with cellular level spatial resolution. Imaging multicellular specimens with subcellular spatial resolution remains a problem with multiview SPIM.

The nonexistence of a perfect light sheet not only limits the 3D imaging ability of SPIM, but also creates a severe yet underappreciated practical problem. Optimization of the light sheet, including its type and dimensions, is required case by case in SPIM depending on the sample to be imaged and the biological question to be studied, as different light sheets balance the 3D imaging ability of SPIM differently^14^. The light sheet is usually determined based on the prior knowledge of the specimen and the biological process to be imaged, and it remains the same during the entire imaging process because a realignment of the microscope, that often takes hours even for SPIM experts, is usually required to change the light sheet. It is not only extremely inconvenient, but also prevents SPIM from adapting itself to reach the optimal imaging performance, because different organelles have different structures and dynamics, and both of these can change in live imaging. Instead, the optimization of SPIM imaging performance would become a closed-loop process if the light sheet could be adjusted in real-time using the immediate imaging result as feedback. More importantly, the understanding of a biological process relies on the spatial and temporal information that can be obtained from the process, but an ideal method that is capable of acquiring both with a high resolution is often unavailable, especially for multicellular specimens. The only option under such circumstances is to compromise either the spatial or the temporal resolution strategically, and only acquire the necessary information needed to understand a biological process. Such ability and flexibility to compromise can only be obtained by adjusting the light sheet in real-time in SPIM. Thus, being able to optimize the excitation light sheet in real-time is another key to improve the 3D live imaging ability of SPIM on multicellular specimens.

Recently, we described a tiling light-sheet SPIM technique (TLS-SPIM) to overcome the SPIM tradeoff between the spatial resolution, optical sectioning capability and FOV by tiling the excitation light sheet^17^. Instead of using a large light sheet to illuminate the entire FOV simultaneously, a small but thin light sheet is tiled quickly within the image plane to illuminate the whole FOV, while only the fluorescence signal generated at centre of each tiled light sheet is used in the final image (Supplementary Figs 2 and 3). As a result, both high spatial resolution and good optical sectioning capability are maintained within a FOV that is much larger than the light sheet itself. Potentially, multicellular specimens could be imaged by TLS-SPIM with either higher spatial resolution, better optical sectioning capability or both compared with regular SPIM. Besides TLS-SPIM, several other methods were also developed to increase the FOV of SPIM with different approaches^18^,^19^,^20^. Unfortunately, the imaging ability of these methods are limited by using either two-photon excitation or optical components with slow dynamic performance, while none of these methods can implement all of the latest SPIM light sheets and optimize the light sheet in real-time.

Convinced by the imaging ability of TLS-SPIM observed in our previous research^17^, we continue to develop and explore the potential of this method to facilitate the study of cell behaviours in multicellular specimens. We present TLS-SPIM with real-time light sheet optimization to address the above problems that limit SPIM in imaging multicellular specimens with subcellular spatial resolution. We extend the 3D live imaging ability of TLS-SPIM by enabling the implementation, tiling and real-time optimization of the latest SPIM light sheets in TLS-SPIM, including the Gaussian, Bessel and Lattice light sheet. We compare the 3D imaging ability of TLS-SPIM in resolving complex structures with regular SPIM methods and verify its 3D live imaging performance on different multicellular specimens. In addition, we introduce a methodology to study cell behaviours in multicellular specimens using TLS-SPIM, which is to image intracellular activities of the cells involved in a multicellular process, identify their intrinsic connections with the cellular behaviours, and determine their impact on the multicellular process. We demonstrate the methodology using *C. elegans* embryo left-right symmetry breaking behaviour as an example.

## Results

### Tiling light sheet selective plane illumination microscopy

Our new TLS-SPIM microscope was designed to satisfy two requirements. First, the microscope can implement the latest SPIM light sheets to use their advantages in different applications, and the light sheet can be adjusted quickly to optimize the TLS-SPIM imaging performance in real-time. Next, the light sheet can be tiled rapidly within the detection image plane to enlarge the FOV without affecting the spatial resolution and optical sectioning capability. To achieve these goals, two binary spatial light modulators (SLM) are implemented in sequence in our TLS-SPIM microscope to modulate the excitation light. The two SLMs are conjugated to the image focal plane and the rear pupil of the excitation objective, respectively (Supplementary Fig. 4). Either a Gaussian light sheet, a Bessel light sheet or a Lattice light sheet can be used in the microscope, and it takes less than a millisecond to either tile or change the light sheet by applying different binary phase maps to the SLMs (Supplementary Figs 5–10, Supplementary Movie 1,2).

### 3D imaging ability in resolving complex structures

**Figure 1:**
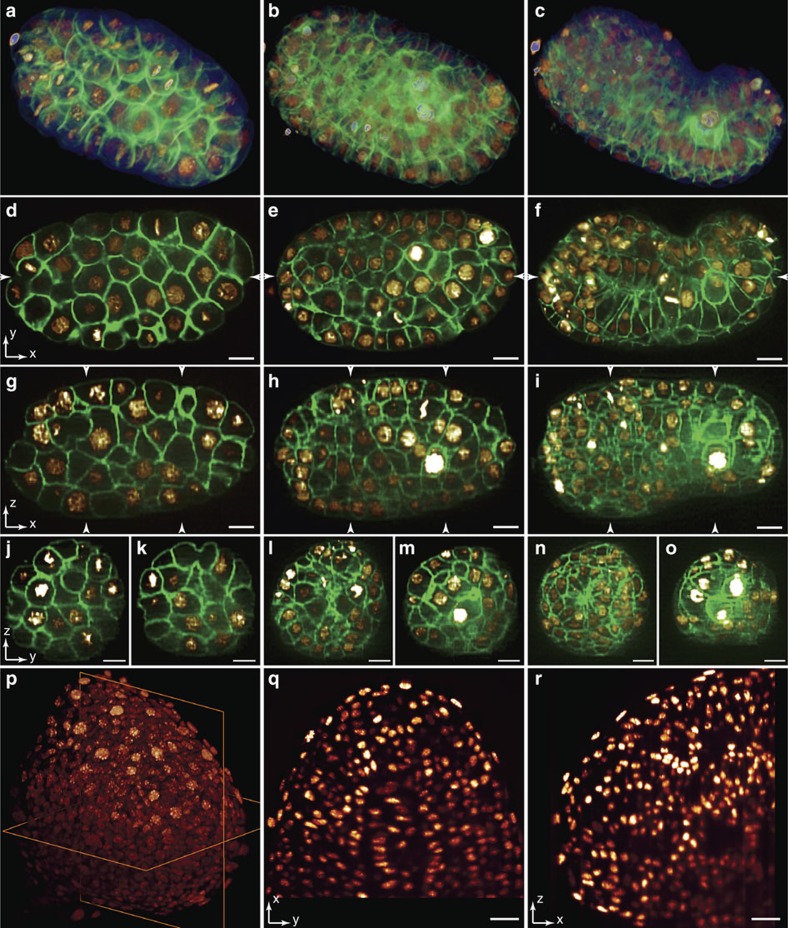
3D imaging ability of TLS-SPIM in resolving complex structures of multicellular specimens. (**a**–**c**) 3D volume renderings of a *C. elegans* embryo (OD95) expressing GFP::PLC∂^PH^ (membrane) and H2B::mCherry (nucleus) at ∼50 cell stage, ∼200 cell stage and bean stage. (**d**–**f**) XY lateral slices through the longitudinal axis of the embryo in a, b and c. (**g**–**i**) YZ axial slices of the embryo in a-c through the positions marked in **d**–**f**. (**j**–**o**) XZ axial slices of the embryo in a-c through the positions marked in **g**–**i**. (**p**) 3D volume rendering of a ∼15 h.p.f. zebrafish embryo tailbud expressing KikGR (nucleus). (**q**–**r**) A XY lateral slice and a XZ axial slice of the tailbud through the planes in p. Scale bars, 5 μm (**d**–**o**) and 20 μm (**q**–**r**).

To examine the ability of TLS-SPIM in resolving complex structures, we imaged a *C. elegans* embryo (OD95) expressing GFP::PLC∂^PH^ (membrane) and H2B::mCherry (nucleus) every 30 min for ∼5 h, from ∼50 cell stage to comma stage until the muscle cells were functioning. A ∼0.7 μm thick, ∼10 μm long Bessel light sheet, generated by scanning a single Bessel beam, was tiled at three positions at 10 μm intervals to image the embryo. The nearly isotropic spatial resolution (∼320 nm lateral, ∼460 nm axial) and good optical sectioning capability enabled the identification of individual cells at most developmental stages (Fig. 1a–o, Supplementary Movie 3,4). Such imaging ability will allow the study of morphogenesis at the necessary subcellular resolution through later stages of embryonic development than was previously possible via other techniques. Specifically, we show that it is difficult to obtain similar results by conventional SPIM with either Gaussian, Bessel or Lattice light sheets (Supplementary Figs 11–14, Supplementary Movie 5,6), as the light sheet must be thicker or the illumination light must be less confined to image the same FOV in regular SPIM (Supplementary Fig. 11). The comparison also shows that the imaging performance of different light sheets varies from sample to sample due to the different sample properties. Instead of sticking to a particular light sheet or method, a more suitable light sheet, including the type, dimensions, tiling number and tiling positions, can always be implemented in TLS-SPIM in real-time with the immediate feedback from the imaging result.

To demonstrate the 3D imaging ability of TLS-SPIM on larger specimens, we imaged the tailbud of a ∼15 h.p.f. nuclei-labelled zebrafish embryo in the next. A ∼1.2-μm thick, ∼30-μm-long Bessel light sheet, which provides ∼320 nm lateral resolution, ∼750 nm axial resolution, was tiled at nine positions at ∼25 μm intervals to image a 0.2 × 0.2 × 0.2 mm volume (Fig. 1p–r, Supplementary Movie 7). Most cell nuclei can be identified and examined with subcellular structural details. Again, the structure was better resolved by TLS-SPIM as compared with regular SPIM attributed to the usage of a thinner light sheet with better light confinement (Supplementary Fig. 16). Nevertheless, as can be observed from the result, the 3D imaging ability of TLS-SPIM on multicellular specimens is still limited by optical aberration and light scattering that affect all light microscopy techniques.

### Live imaging performance

**Figure 2:**
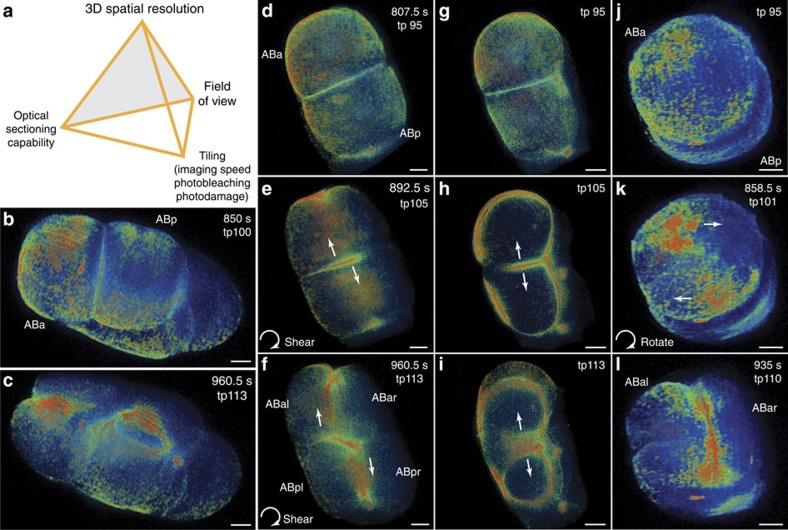
3D live imaging performance of TLS-SPIM. (**a**) The fundamental debate of TLS-SPIM. Spatial resolution and optical sectioning capability are traded for a large FOV in regular SPIM, while the temporal resolution instead can be traded in TLS-SPIM. (**b**–**c**) 3D volume renderings of a 4 to 6 cell stage *C. elegans* embryo (LP162) expressing GFP::NMY-2 (myosin II) at two time points of ∼2 min apart. (**d**–**i**) Orthogonal views of the GFP-tagged myosin II particles distribution in the ABa and ABp cells at prophase, anaphase and cytokinesis, showing the torque generated by the unaligned contractile rings of the ABa and ABp cells. White arrows mark the direction of the pulling forces generated by both contractile rings that produce the torque. (**j**–**l**) Additional views from the top of the Aba cell at prophase, anaphase and cytokinesis, showing the torque generated by the counter flow of asymmetrically distributed myosin II particles on the ABa cell contractile ring. White arrows mark the direction of pulling forces generated by the two myosin II particle groups. (Different color maps were used for clearer visualization). Scale bars, 5 μm.

Due to the light sheet tiling in TLS-SPIM, each image plane is illuminated multiple times equivalent to the number of tiling positions, which raises concerns of slower imaging speed and higher photobleaching and photodamage. Figure 2a depicts the fundamental debate of TLS-SPIM. In regular SPIM, either the spatial resolution, optical sectioning capability or both must be traded for a larger FOV, while in TLS-SPIM, the temporal resolution can be traded as a substitute for FOV by tiling the light sheet. The decision of whether to sacrifice the temporal resolution, and how much to sacrifice will depend on the biological question to be answered. In addition, the problems caused by light sheet tiling are fully predictable and controllable by limiting the number of tiling positions.

**Figure 3:**
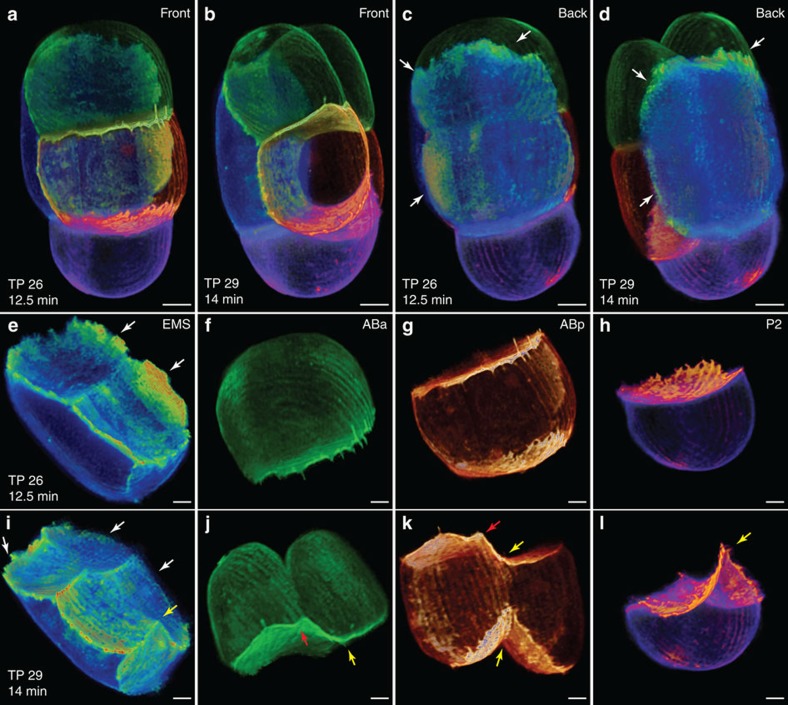
Left-right symmetry breaking of the *C. elegans* embryo at 4-6 cell stage. (**a**–**d**) 3D volume renderings of a *C. elegans* embryo (OD95) viewed from the front (**a**,**b**) and back (**c**,**d**) at two time points of 1.5 min apart at the 4 to 6 cell stage (Cells are segmented and only the cell membrane is rendered). All cells are segmented and rendered in different colours for clearer visualization. (**e**–**h**) 3D volume renderings of the segmented EMS, ABa, ABp and P2 cells at the earlier, and (**i**–**l**) the later time point, showing the intracellular activities and interactions of different cells. The white, red and yellow arrows mark the activities related to the EMS cell retreating, the ABa cell contractile ring and the ABp cell contractile ring. Scale bars, 5 μm (**a**–**d**), 3 μm (**e**–**l**).

**Figure 4:**
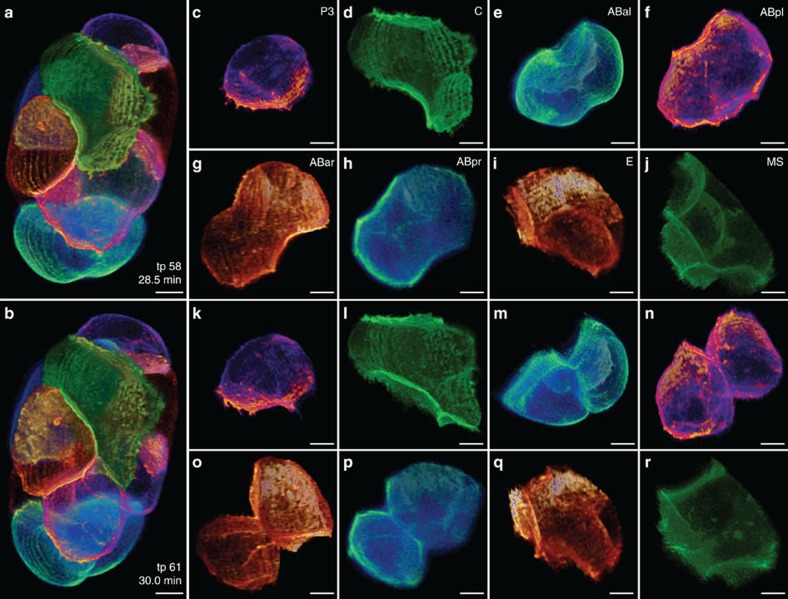
A segmented *C. elegans* embryo at 8–12 cell stage. (**a**,**b**) 3D volume renderings of a *C. elegans* embryo (OD95) at two time points of 1.5 min apart at the 8- to 12-cell stage (Only the cell membrane is rendered). All cells are segmented and rendered in different colors for clearer visualization. (**c**–**j**) 3D volume renderings of the segmented P3, C, ABal, ABpl, ABar, ABpr, E and MS cells at the earlier, and (**k**–**r**) the later time point. Scale bars, 5 μm.

To evaluate the live imaging performance of TLS-SPIM, we imaged endogenously labelled myosin II particle activities in a live *C. elegans* embryo (LP162) every 8.5 s for 150 time points (Fig. 2, Supplementary Movie 8). A Bessel light sheet, generated by scanning an incoherent seven Bessel beam array, was tiled at three positions to image the embryo (Supplementary Movie 1). Both the spatial and temporal resolution of our results are sufficient to track and analyze the myosin II particle movements in 3D, by which the behaviour of the actomyosin network and its impact on embryonic cells can be studied. In another example, we imaged a *C. elegans* embryo (OD95) with the same light sheet every 30 s in two channels for 167 time points (Supplementary Movie 9). Both high spatial resolution and good SNR were maintained through the process, and the embryo hatched successfully in the end (Supplementary Fig. 17, Supplementary Movie 10). The result shows comparable spatial resolution, SNR, imaging speed and much longer imaging time compared with the results obtained from similar specimens imaged by Bessel SPIM and Lattice light-sheet microscopy in previous publications^6^,^7^. The result allows us to extract the dynamic information of every individual cell and analyze their relationship over developmental time, facilitating both visualization and understanding of cell behaviours as embryonic development progresses (Figs 3 and 4, Supplementary Movie 11,12). Clearly, TLS-SPIM performs well for 3D live imaging in terms of the spatial resolution, SNR, imaging speed, photobleaching and photodamage when the tiling number is kept low.

### Correlating cellular behaviours with intracellular activities

A new methodology can be established to study cellular behaviours in multicellular specimens with the dynamic information of intracellular activities acquired by TLS-SPIM. One area of active interest has been the identification of the left-right symmetry breaking mechanism of *C. elegans* embryos at the four- to six-cell stage^21^,^22^,^23^,^24^. TLS-SPIM can provide high spatial and temporal resolution information of different intracellular activities involved in the process, such as the actomyosin network behaviour and the deformation of each individual cell, implicating the mechanical mechanism that drives symmetry breaking. We identified three intracellular activities that could have major contributions to the symmetry breaking behaviour mechanically. First, the torque generated by the unaligned contractile rings of the ABa and ABp cells during mitosis could shear the ABar and ABpr to ABal and ABpl cells (Fig. 2d–i, Supplementary Movie 13). Second, the simultaneous relative rotation of their daughter cells, ABal to ABar and ABpl to ABpr, could be caused by the torque generated by the counter flow of asymmetrically distributed myosin II particles on the contractile ring of ABa cell, which is consistent with previous observation^23^ (Fig. 2j–l, Supplementary Movie 13). Third, the retreating of the EMS cell, entering mitosis afterwards, could assist the symmetry breaking behaviour and the positioning of all daughter cells by pulling the neighbour cells (Fig. 3, Supplementary Movie 11). Our observations not only provide evidence to verify some of the previous hypotheses^23^,^24^, but also suggest a new mechanism to explain the behaviour. Based on these observations, more specific experiments can be designed, quantitative image analysis tools and mechanical modelling methods can be introduced to analyze and understand the biochemical and biophysical mechanism of the symmetry breaking behaviour. As demonstrated, with the advanced 3D live imaging ability of TLS-SPIM, instead of treating different cell behaviours isolatedly, a methodology of imaging and analysing intracellular activities of different cells and finding their intrinsic connections can be applied to study cell behaviours in multicellular specimens.

The methodology can certainly be applied to later stage *C. elegans* embryos and other multicellular specimens with the 3D imaging ability of TLS-SPIM. For instance, by examining all cells of the same embryo at two time points of 1.5-min apart at the 8- to 12-cell stage (Fig. 4, Supplementary Movie 12), we noticed that both the shape and position of the C cell, E cell and MS cell changed following the mitosis of the ABal, ABar, ABpl and ABpr cells, that divided almost synchronously. The positions of ABal, ABar, ABpl and ABpr daughter cells also seem affected by the synchronized mitotic processes of these cells. The consistent observations of aggressive cell position rearrangement during synchronized mitotic processes through embryo development also suggest that the contractile rings of synchronized mitotic cells have an important role in cell positioning and morphogenesis of early stage *C. elegans* embryos. More imaging and quantitative analysis on different intracellular activities that are related to force generation are required to understand these behaviours completely.

### A compromised live imaging solution

**Figure 5:**
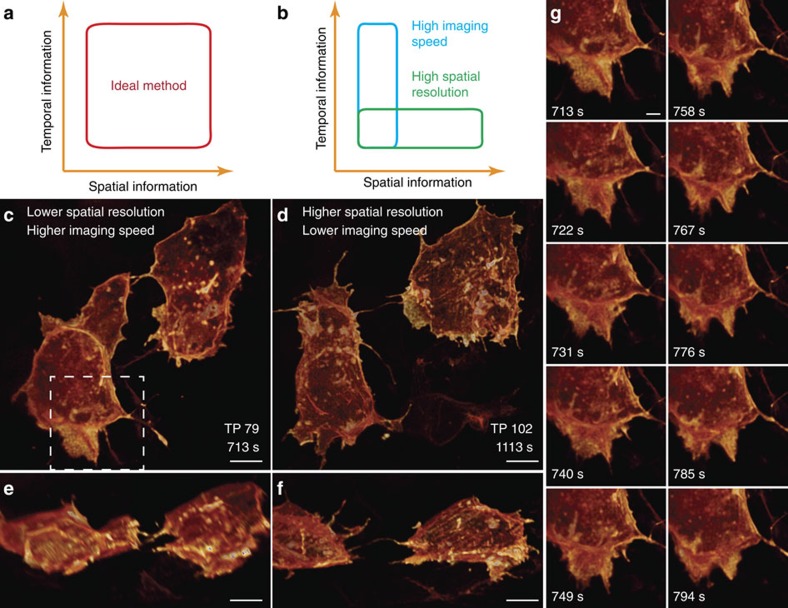
3D imaging by TLS-SPIM with real-time light sheet optimization. (**a**) An ideal imaging method with both high spatial resolution and imaging speed acquires spatial and temporal information simultaneously. (**b**) A compromised solution acquires spatial information and temporal information separately by quick switching between a high spatial resolution, low imaging speed mode and a low spatial resolution, high imaging speed mode. (**c**,**d**) 3D volume renderings of two mesodermal cells in the tailbud of a ∼15 hpf zebrafish embryo imaged in a low spatial resolution, high imaging speed mode and a high spatial resolution, low imaging speed mode. (**e**,**f**) Orthogonal views of the cells in c and d. (**g**) Ten continuous time points of the selected area in c imaged in the low spatial resolution, high imaging speed mode, showing the lamellipodia activities of the mesodermal cell. Scale bars, 5 μm (**c**–**f**), 2 μm (**g**).

TLS-SPIM requires more tiling positions to further increase the FOV and maintain the same spatial resolution and optical sectioning capability, making it less ideal for high-resolution live imaging of large multicellular specimens. As no imaging technique can achieve submicron 3D spatial resolution and high imaging speed simultaneously on specimens of hundreds of microns or larger, we developed a compromised solution to handle this problem, which is to acquire the spatial and temporal information of a biological process separately, namely, alternating the 3D imaging between a high spatial resolution, low imaging speed mode and a low spatial resolution but high imaging speed mode (Fig. 5a,b). In this way, the results acquired in different modes could still provide sufficient spatial and temporal information to understand the imaged process, as long as the switching between different modes is fast enough. Our TLS-SPIM microscope enables rapid switching by adjusting the excitation light sheet in less than a millisecond, realized by changing the phase maps applied to the binary SLMs used in the microscope, (Supplementary Figs 5–10). To demonstrate this idea, we imaged cell migration of mosaically labelled mesodermal cells expressing a red fluorescent protein (Cherry)-tagged membrane marker in the tailbud of a ∼15 h.p.f. zebrafish embryo. The region of interest (80 × 80 × 50 μm) was imaged with ∼320 nm lateral resolution, ∼750 nm axial resolution every ∼9 s, and with ∼320 nm lateral resolution, ∼450 nm axial resolution every minute alternately. By this method, both the 3D structure and the dynamics of the migrating cells can be clearly visualized (Fig. 5c–g, Supplementary Movie 14,15,16). A notable difference between the imaged cells and cells migrating on 2D substrates is the absence of membrane ruffling behaviour, despite the similar lamellar protrusions^7^, which could be caused by more strict constraints on cell membrane by neighbour cells in multicellular specimens. As shown, a reasonable understanding of a biological process can still be obtained by compromising the imaging ability flexibly when an ideal method is not available. Such ability is especially valuable in imaging large multicellular specimens as they are more difficult to image and the cellular behaviours are more complicated in such specimens.

## Discussion

TLS-SPIM with real-time light-sheet optimization not only offers advanced 3D live imaging ability, allows new methodology to be applied to understand cell behaviours in multicellular specimens, but also enables the delivery of such imaging ability and analysis method to general SPIM users with its special instrument design, as there is no hardware change required to operate the microscope after the initial alignment, including the light-sheet optimization, tiling and imaging mode switching. A deep understanding of the TLS-SPIM technique itself is not necessary for general users to use this technique, the most suitable imaging condition, including the light sheet and its dimensions, tiling numbers, positions and imaging modes can be determined solely based on the sample features, desired spatial resolution, SNR, imaging speed, FOV and the acquired images. The phase maps to be applied to the SLMs can be calculated automatically and applied accordingly. On the other hand, TLS-SPIM can take the advantages of most previously developed SPIM techniques rather than conflict with any of them, such as the different light sheets and the multiview SPIM configuration. Altogether, TLS-SPIM with real-time light-sheet optimization will make SPIM much more feasible and favourable to general biologists.

Two perceived problems are still limiting the 3D imaging ability and application of SPIM. First, SPIM is also limited by optical aberration and light scatting as other optical imaging techniques, and the problem is doubled in SPIM due to the separated illumination and detection. An effective combination of SPIM and adaptive optics without heavily affecting its live imaging performance is desired. After all, how well SPIM performs relies on how well the excitation light is confined. Next, SPIM is unique due to the enormous spatial and temporal information that can be collected with it. However, appropriate quantitative image analysis tools are not yet widely available to general users to make most of the collected information useful. On the other hand, the development of appropriate image analysis tools itself faces many challenges at the same time, because of the huge variety of the biological questions, the imaging ability difference of the different SPIM techniques, and the lack of clear indications of what kind of quantitative information is desired and how the information can be used. The gap between biologists, physicists and computer scientists must be filled to have this problem solved. Ultimately, moving cell biology research from 2D to 3D is inspiring but still challenging. The solutions to the above problems will perhaps determine how much impact SPIM can make in accelerating the transition.

## Methods

### Design considerations

#### The key tradeoff in SPIM

Axial resolution, optical sectioning capability and FOV are three key factors affecting the 3D imaging ability of SPIM. These factors are only determined by the intensity profile of the excitation light sheet in regular SPIM with a given detection NA (Supplementary Fig. 1). The axial resolution, defining the smallest axial separation SPIM can resolve in theory, relies on the thinnest axial component of the excitation light sheet in real space, which corresponds to the highest axial frequency of the light sheet optical transfer function (OTF) in frequency space. The optical sectioning capability measures the ability of SPIM to suppress out-of-focus fluorescence background. It determines whether the theoretical spatial resolution, especially the axial resolution, can be achieved with high-enough SNR in practice. The optical sectioning capability of SPIM depends on the percentage of the illumination light confined within the detection depth of focus in real space, and it is also reflected by how quickly the light-sheet OTF amplitude rolls off to zero along the axial direction in frequency space^13^,^14^.

Aside from the excellent axial resolution and optical sectioning capability of SPIM, FOV is a major limiting factor of SPIM due to the optical configuration of SPIM. In general, it is limited by the length of the excitation light sheet along the light propagation direction, where the light sheet intensity profile remains uniform. For this reason, the light sheet length must be increased to enlarge the SPIM FOV for large specimens. However, no matter what kind of light sheet is used, the light sheet either becomes thicker or the illumination light is less confined as its length increases due to the diffraction of light. As a result, the size of SPIM FOV always goes opposite to the SPIM axial resolution and optical sectioning capability. Therefore, the relationship between SPIM axial resolution, optical sectioning capability and FOV represents a key tradeoff in SPIM, and this tradeoff cannot be overcome by regular SPIM due to the diffraction of light. TLS-SPIM provides a solution to this problem.

#### Real-time optimization of the excitation light sheet

Because of the tradeoff in SPIM, no light sheet is suited for all specimens. Optimization of the light sheet is required in SPIM, and the benefit of SPIM is not guaranteed unless an appropriate light sheet is selected based on the specimen to be imaged and the question to be answered. Different light sheets have been developed for SPIM imaging. Typical light sheets include Gaussian light sheets, Bessel light sheets and Lattice light sheets and so on^1^,^2^,^3^,^4^,^5^,^6^,^7^,^15^. For light sheets of comparable sizes, Gaussian light sheets have the most restricted light confinement, but they are usually too thick to obtain submicron axial resolution. Bessel light sheets contain a much thinner central peak that enables high axial resolution to be obtained in theory, but the optical sectioning capability is sacrificed due to the poor illumination light confinement. Lattice light sheets reach a better balance between light sheet thickness and light confinement compared with the previous two by using a coherent Bessel beam array, but it is more sensitive to optical aberration and light scattering (Supplementary Figs 11,14,15)^13^,^14^.

On the other hand, SPIM lowers the photobleaching and photodamage compared with conventional fluorescence microscopy techniques. Obviously, light sheets confining more excitation light within the detection depth of focus produce less photobleaching and photodamage. Nevertheless, the photodamage of SPIM depends not only on the light sheet intensity profile, but also on how it is generated. In SPIM, the excitation light sheet can be either a real one, existing simultaneously across the entire illumination field, or a virtual one, created by scanning an excitation beam or dithering a beam array. As a consequence, for light sheets of the same intensity profile, a real light sheet has a lower peak intensity than a virtual one created by dithering a beam array or scanning a single excitation beam. Although it has not been studied systematically due to the complexity of a fair comparison and different natures of different specimens, lower excitation peak intensity is usually preferred to decrease the photodamage. Using a light sheet with lower peak intensity should always be considered when photodamage becomes a major problem^6^,^7^. For the above reasons, to adjust the light sheet in real-time based on the immediate feedback from the imaging result is important to improve the 3D live imaging ability of SPIM and a general guidance of how to choose SPIM light sheet was described in previous publications^13^,^14^.

We conceived our research and constructed our microscope accordingly.

### Microscope configuration

The schematic diagram of the microscope is shown in Supplementary Fig. 4. CW lasers with excitation wavelengths of 488 and 561 nm are used for linear excitation (Coherent, Sapphire LP, 200 mW). Laser beams from both lasers are expanded to a common 1/e^2^ beam diameter of 1.5 mm and combined into a single co-linear beam using a LaserMux dichroic beam combiner. The combined laser beam is sent to an acousto-optical tunable filter (AOTF, AA Opto-Electronic, nAOTFnC-400.650-TN) used to select one or more wavelengths and modulate the laser beam intensity. The laser beam is directed to one of the two separated optical paths after the AOTF and a half-wave plate HWP1.

The microscope is operated in two modes by sending the laser beam to the two separated optical paths. In the first mode (Supplementary Fig. 4a), a binary SLM, SLM2, conjugated to the rear pupil of the excitation objective is used to generate a single excitation beam or an incoherent beam array. A virtual excitation light sheet is generated by scanning the excitation beam or dithering the excitation beam array using a mirror galvanometer. SLM2 is also used to tile the excitation light sheet by changing the excitation beam/beam array position along the beam direction. In the second mode (Supplementary Fig. 4b), Lattice light sheets are used for SPIM imaging. Two binary SLMs, SLM1 and SLM2 are implemented in sequence, in which SLM1 is conjugated to the image focal plane of the excitation objective and SLM2 is still conjugated to the rear pupil of the excitation objective. A Lattice light sheet is generated by applying the corresponding phase maps to both SLM1 and SLM2, and the light sheet is tiled by SLM2 in the same way as that in the first mode.

In the second mode, the laser beam is expanded to 15 mm in vertical direction by a pair of cylindrical lenses (focal length CL1=25 mm, CL2=250 mm) and sent to SLM1. The SLM assembly consisted of a polarizing beam splitter cube, a half-wave plate and a 1,280 × 1,024 pixel binary SLM (Forth Dimension, SXGA-3DM). The SLM1, conjugated to the excitation objective image focal plane, is imaged to the second identical binary SLM assembly, SLM2, through lens L5=500 mm. After that, the laser beam is sent to the beam scanning assembly consisted of a pair of galvos (Cambridge Technology, 6215HP-1HB) for laser beam and light-sheet scanning. Both galvos are conjugated to SLM2, through relay lenses L6=150 mm, L7=75 mm and conjugated with each other through relay lenses L8=100 mm, L9=100 mm, and finally conjugated to the rear pupil of the excitation objective (Nikon, CFI Apo 40XW NIR 0.8 NA) through relay lenses L10=75 mm and L11=150 mm. An optical slit was placed at the focal plane of lens L6, to block the undesired diffraction orders generated by SLM2. The detection objective (Nikon, CFI Apo 40XW NIR 0.8 NA), mounted on an objective scan piezo (PI, P-724), is placed orthogonal to excitation objective. The emitted fluorescence is collected through the detection objective and imaged onto a sCMOS camera (Hamamatsu, Orca Flash 4.0) by tube lens L12=225 mm. In the first operation mode, the laser beam is directed to the other optical path by flip mirrors FM1 and FM2. The laser beam is expanded to 6 mm diameter by relay lenses L3=25 mm and L4=100 mm and sent to SLM2 after that. The laser beam followed the same optical path as that in the other mode after SLM2. Manual operation is required to switch the microscope between the two modes. The flip mirrors FM1 and FM2 can be replaced by galvos to allow fast switching between the two operation modes. A wildfield optical path used to find samples before SPIM imaging is not shown in the schematic diagram. The control system and hardware are the same as what reported in the previous publications^5^,^6^,^13^. The sCMOS camera triggers are used to synchronize the operation of the SLMs, galvos, objective piezo and camera itself.

### Microscope working principle

The microscope was designed to satisfy two requirements. First, the excitation light sheet can be tiled quickly within the detection image plane to enlarge the FOV without affecting the axial resolution and the optical sectioning capability. Next, both the type and the dimension of the light sheet can be changed quickly to optimize the SPIM imaging performance in real-time.

Tiling of the excitation light sheet can be realized by adding a variable spherical phase map to the excitation light wavefront at a plane conjugated to the excitation objective rear pupil. It can be achieved by using either a SLM, a deformable mirror, or a focus variable lens. We use a binary SLM because it is flexible, easy to control and the light sheet can be positioned fast and accurately by applying different phase maps. The applied phase map can be refreshed in less than a millisecond (∼3.2 K Hz rate), which enables both fast tiling and real-time optimization of the light sheet. The method of tiling the light sheet with a binary SLM by applying a spherical phase map was described in details in our previous publication^17^.

The excitation light sheet used in SPIM is usually generated by scanning a single-excitation beam or dithering a beam array. The intensity profile of light sheet is adjusted by changing the beam or the beam array used to create the light sheet. It can be realized by modifying the intensity and phase profile of the excitation laser beam. For example, circular laser beams of different diameters, corresponding to different excitation NAs (NA_exc_) can be used to create Gaussian beams of different dimensions. Annular laser beams of different inner and outer diameters, corresponding to different inner and outer excitation NA (NA_ID_, NA_OD_), can be used to create different Bessel beams. A metal coated quartz mask containing transmissive patterns of different geometries is usually used to shape the laser beam intensity profile and create these beams^5^,^6^,^7^,^13^,^14^. However, the number of options is limited and realignment is required to switch between different beams in such a way.

In our microscope, SLM2 is also used to modify the intensity and phase profile of the excitation laser beam to adjust the light sheet intensity profile in real-time besides tile the light sheet. The method is shown in Supplementary Fig. 5. The SLM2 is separated to two areas, retain area and abandon area. A high frequency binary phase grating is applied to the abandon area, so that the incident light falling on the abandon area is diffracted to different directions from the light falling on the retain area. The abandoned light is lost or blocked by the optical slit afterwards. The retained light is allowed to propagate along the designed optical path. For example, the annular amplitude mask in Supplementary Fig. 5a used to generate a Bessel beam can be replaced by applying the phase map shown in Supplementary Fig. 5b to SLM2. It is calculated by multiplying a reverse binary amplitude mask, shown in Supplementary Fig. 5c, and a high frequency binary phase grating (two pixels per period) shown in Supplementary Fig. 5d. In this way, the laser beam intensity profile can be modified by adjusting the shape and size of the retain area. Further modification can be done by controlling the phase within the retain area of SLM2. Most excitation beams/light sheets can be generated by this method, and we developed specific solutions to generate either a single Gaussian/Bessel beam, an incoherent Gaussian/Bessel beam array or a coherent Bessel beam array in our microscope, so that both Gaussian light sheets, Bessel light sheets and Lattice light sheets can be used in our microscope. The details are discussed below.

### Single excitation beam

The microscope is operated in the first mode, and only SLM2 is used. The excitation beam and its dimension are controlled by shape and size of the retain area on SLM2. The tiling of the light sheet is realized by applying a binary spherical phase map to the retain area. Extra diffraction orders are blocked by the optical slit. The details to generate the binary spherical phase map is discussed in our previous publication^17^. For example, a Bessel beam (Supplementary Fig. 6d, NA_OD_=0.35, NA_ID_=0.14) was generated by applying the phase map in Supplementary Fig. 6a. The same beam was moved to the right side of the previous position (Supplementary Fig. 6e) by applying the phase map in Supplementary Fig. 6b. A thicker and longer Bessel beam (Supplementary Fig. 6f, NA_OD_=0.2, NA_ID_=0.05) was generated on the left side of the initial beam position by applying the phase map in Supplementary Fig. 6c.

### Incoherent beam array

The microscope also works in the first mode to generate an incoherent excitation beam array. A light sheet with lower peak intensity can be generated by dithering the beam array. An incoherent beam array with beams of roughly equal intensity can be generated by using a Dammann grating^25^,^26^, which is a periodic binary phase pattern. In the meantime, the beam array can be tiled by superimposing a continuous spherical phase map to the Dammann grating. A binary phase map to generate the beam array at a desired position is obtained by resetting the pixel values of the superimposed phase map to 0 and π. Values between 0 and π are set to 0, and values between π and 2π are set to π. The final binary phase map applied to SLM2 is obtained by replacing the phase map of the abandon area with the high frequency grating pattern. Supplementary Figure 7a and 7c show two Dammann gratings that generate a 7 beam array and a 21 beam array, respectively. Supplementary Figure 7b and 7d show a full period of each grating. Supplementary Figure 7e and 7f show two continuous phase maps to be superimposed to the Dammann gratings to move a beam array to different positions. Supplementary Figure 7g and 7h show two binary phase maps applied to SLM2 to generate a Bessel beam array at two different positons (Fig. 4k,l, NA_OD_=0.35, NA_ID_=0.14). Supplementary Fig. 7i and j show two phase maps applied to SLM2 to generated a 21 beam Bessel beam array at two different positons (Supplementary Fig. 7m and 7n, NA_OD_=0.5, NA_ID_=0.14) (Supplementary Movie 1).

However, this method has several limitations due to the limited SLM pixels. To create a beam array of *N* beams using a Dammann grating, 2 × int[(*N*−1)/4]+2 transition points are required per grating period^26^, while the minimum distance between any two adjacent transition points must be larger than one pixel to allow the grating pattern to be applied to the SLM. In consequence, the pixel number per grating period has to be increased as the number of beams increases, which decreases the corresponding beam array period at the same time. On the other hand, the beam array period has to be large enough to make the beam array wide enough to cover the desired FOV and avoid the interference between different beams, because the Dammann grating doesn't control the phase relationship between different beams. For above reasons, it is difficult to generate a beam array of more than 21 beams by this method, and the selection of beam array period corresponding to each beam number is also limited.

### Coherent bessel beam array

TLS-SPIM works in the second mode to use a tiling Lattice light sheet. Both SLM1 and SLM2 are used to generate and tile a coherent Bessel beam array in this mode. As reported in previous research from Betzig's lab^7^, a binary phase map generated from the desired beam array amplitude and phase profile is applied to a binary SLM that conjugated to the image focal plane of the excitation objective. The SLM is imaged to a transmissive annular pattern on a photo mask, which is conjugated to the excitation objective rear pupil to remove the undesired diffraction orders. A similar method is used in our microscope to generate a coherent Bessel beam array except that SLM2 is used to replace the photo mask and tile the Lattice light sheet. Supplementary Figure 8a shows a binary phase map applied to SLM1 to generate a coherent excitation beam array. The high frequency grating pattern is also used to remove the undesired illumination light. Supplementary Figure 8b and 8c show two phase maps applied to SLM2 (NA_OD_=0.35, NA_ID_=0.14) to shape the excitation laser beam and tile the coherent Bessel beam array. Supplementary Figure 8d and 8e show the corresponding results by applying Supplementary Fig. 8a to SLM1, Supplementary Fig. 8b to SLM2 and Supplementary Fig. 8a to SLM1 and Supplementary Fig. 8c to SLM2. Supplementary Figure 8f shows the intensity profile of the laser beam at the excitation objective pupil plane, and Supplementary Figure 8g shows the measured cross intensity profile of the generated coherent beam array at the excitation objective focus (Supplementary Movie 2).

A beam array that is more close to an incoherent beam array can also be generated in this mode with the same method by increasing the beam array period (Supplementary Fig. 9). The beam array period and the number of beams can be controlled with more flexibility in this mode compared to the previous one, despite the more complicated optical system and more laser power loss due to the use of two SLMs.

### Microscope alignment and calibration

The microscope alignment is similar to what reported in our previous publication^13^. The binary phase maps used to generate different excitation beams/beam arrays at different positions in the two working modes were calculated in MATLAB in advance and calibrated in fluorescent dye solution. All phase maps were loaded into the corresponding SLM prior to experiments. Selected phase maps are loaded in sequence and synchronized by the sCMOS camera during 3D imaging. Potentially, phase maps corresponding to different light sheets can be calculated *in situ* with the given parameters of the optical system components after the calibration.

### Image analysis

3D image stacks collected at different light sheet tiling positions were stitched together according to the length and tiling positions of the light sheet. The combined image stack was deconvolved using the Richardson-Lucy 3D deconvolution algorithm in MATLAB^13^. The point spread function corresponding to the light sheet was collected by imaging an isolated 100 nm fluorescent particle. The deconvolved result was filtered with low pass filters to remove noises beyond the microscope OTF. Cell segmentation was performed using Matlab and Amira. Analysed image data were rendered in 3D with Amira for display. More accurate image registration method to combine different image stacks is under development.

### Sample preparation

*C. elegans* embryos were dissected from adult worms. Dissected *C. elegans* embryos were placed directly on top of poly-D-lysine-coated coverslips for imaging. The following strains were used: OD95 ltIs37 [pAA64; *pie-1*::mCherry::HIS-58+*unc-119*(+)] IV. ltIs38 [pAA1; *pie-1*::GFP::PLC∂^PH^+*unc-119*(+)], LP162 *nmy-2*(cp13[*nmy*-2::gfp+LoxP]) I. RW10029 zuIs178 *[his-72(1kb 5' UTR)::his-72::SRPVAT::GFP::his-72* (1KB 3′ UTR)+5.7 kb *Xba*I - *Hin*dIII *unc-119*(+)]. stIs10024 [*pie-1*::H2B::GFP::pie-1 3′ UTR+*unc-119*(+)].

Nuclei of zebrafish embryos were labelled by ubiquitous expression of nuclear localized Kikume green/red photoconvertible protein (NLS-KikGR). Membrane labelling was achieved by injecting 25 picograms of a reporter plasmid at the 1-cell stage. The plasmid consisted of a 1kb fragment of the *ntla* promoter driving expression of membrane localized Cherry, in order to enrich for expression in mesodermal cells. Injected plasmid DNA is inherited mosaically in zebrafish embryos, causing cells to be scatter-labelled. Embryos were imaged in their chorions at the 6-12 somite stages, focusing on the posteriorly localized embryonic structure of the tailbud. To mount the zebrafish embryo, a drop of 1% low melting temperature agarose gel was dropped on top of a cleaned coverslip, and the zebrafish embryo was place on top of the melted Agarose drop afterwards. The embryo is ready for imaging after the agarose gel is solidified. All embryos were imaged in water at 20 °C.

## Additional information

**How to cite this article**: Fu, Q. *et al*. Imaging multicellular specimens with real-time optimized tiling light sheet selective plane illumination microscopy. *Nat. Commun.* 7:11088 doi: 10.1038/ncomms11088 (2016).

## Supplementary Material

Supplementary Information Supplementary Figures 1-17

Supplementary Movie 1 An incoherent seven Bessel beam array tiled at seven positions in dye solution (NAOD=0.35, NAID=0.14).

Supplementary Movie 2 A coherent Bessel beam array tiled at seven positions in dye solution (NAOD=0.35, NAID=0.14).

Supplementary Movie 3 Volume rendering and orthogonal slices of a C. elegans embryo (OD 95) imaged by TLS-SPIM every 30 minutes for 5 hours (time point 1-5). Upper left: 3D volume rendering, upper right: XY slice, lower left: XZ slice, lower right: YZ slice. Associated with Figure 1 (NAOD=0.35, NAID=0.14, tiled at three positions).

Supplementary Movie 4 (Continue from Supplementary Movie 3) Volume rendering and orthogonal slices of a C. elegans embryo (OD 95) imaged by TLS-SPIM every 30 minutes for 5 hours (time point 6-10).

Supplementary Movie 5 XZ axial slices of three C. elegans embryos (OD95 and RW10029) imaged by regular SPIM (left, middle) and TLS-SPIM (right). Associated with Supplementary Figure 12 and 13.

Supplementary Movie 6 XZ axial slices of two C. elegans embryos (OD95) imaged by regular Lattice light sheet microscopy (left), TLS-SPIM with a tiling Lattice light sheet (middle) and TLS-SPIM (right) with a tiling light sheet generated by dithering an incoherent Bessel beam array. Associated with Supplementary Figure 14.

Supplementary Movie 7 Volume rendering and orthogonal slices of a ~15 hpf nuclei-labeled zebrafish embryo tailbud. Upper left: 3D volume rendering, upper right: XY slice, lower left: YZ slice, lower right: XZ slice. Scale bar 20 μm. Associated with Figure 1 and Supplementary Figure 16.

Supplementary Movie 8 Volume renderings of a C. elegans embryo (LP162) expressing GFP-tagged myosin II viewed from different directions. Associated with Figure 2 (NAOD=0.35, NAID=0.14, tiled at three positions).

Supplementary Movie 9 Volume renderings of a C. elegans embryo (OD95) imaged every half-minute in two colors, viewed from two orthogonal directions. Left: lateral, right: axial. Scale bar 10 μm. Associated with Figure 3, 4 and Supplementary Figure 17 (NAOD=0.35, NAID=0.14, tiled at three positions).

Supplementary Movie 10 XZ axial slices of the same C. elegans embryo (Supplementary Movie 8) at the selected time points (green channel). Associated with Figure 3, 4 and Supplementary Figure 17.

Supplementary Movie 11 Volume rendering of the same C. elegans embryo (Supplementary Movie 8) at the first 61 time points with all cells segmented and viewed from four orthogonal directions. Associated with Figure 3, 4.

Supplementary Movie 12 Volume rendering of all cells of the same C. elegans embryo (Supplementary Movie 8) at two selected time points of 1.5 minutes apart at the 8 to 12 cell stage. Associated with Figure 4.

Supplementary Movie 13 Volume renderings of the ABa and ABp cells of the same embryo (Supplementary Movie 7) showing the unaligned contractile rings during cytokinesis and the counter flow of two myosin II particle groups on the ABa cell. Different colormaps were used in left panels and right panels. Associated with Figure 2.

Supplementary Movie 14 Volume rendering of two mesodermal cells in a ~15 hpf zebrafish embryo tailbud imaged by TLS-SPIM alternated between a low spatial resolution, high imaging speed mode (NAOD=0.2, NAID=0.05, tiled at two positions) and a high spatial resolution, low imaging speed mode (NAOD=0.35, NAID=0.14, tiled at seven positions). Upper left and lower left: volume renderings from different directions, upper right and lower right: lateral and axial max projections. Associated with Figure 5.

Supplementary Movie 15 Volume renderings of the two mesodermal cells imaged in the two modes showing the different spatial resolution. Associated with Supplementary Movie 13 and Figure 5.

Supplementary Movie 16 Volume renderings of another two mesodermal cells in a ~15 hpf zebrafish embryo tailbud imaged by TLS-SPIM alternated between the two modes.
