# Supplementary material for: Imaging multicellular specimens with real-time optimized tiling light-sheet selective plane illumination microscopy
Source: Nat Commun. 2016 Mar 23;7:11088. doi: 10.1038/ncomms11088 (PMC4814582; doi:10.1038/ncomms11088)
Supplement: Supplementary Information — Supplementary Figures 1-17 [file ncomms11088-s1.pdf]

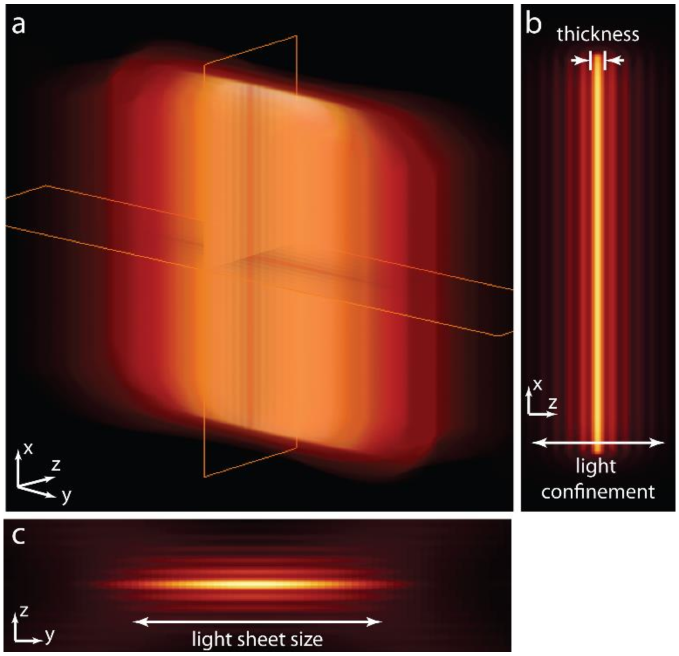

Supplementary Figure 1. The key tradeoff in SPIM. With a given detection NA, the excitation light sheet thickness, light confinement and light sheet size determine the axial resolution, optical sectioning capability and FOV respectively. (a) A simulated Bessel light sheet rendered in 3D is used as an example to depict the problem. (b) XZ cross section intensity profile of the light sheet showing the thickness and light confinement of the light sheet. (c) YZ cross section intensity profile of the light sheet showing the size of the light sheet.

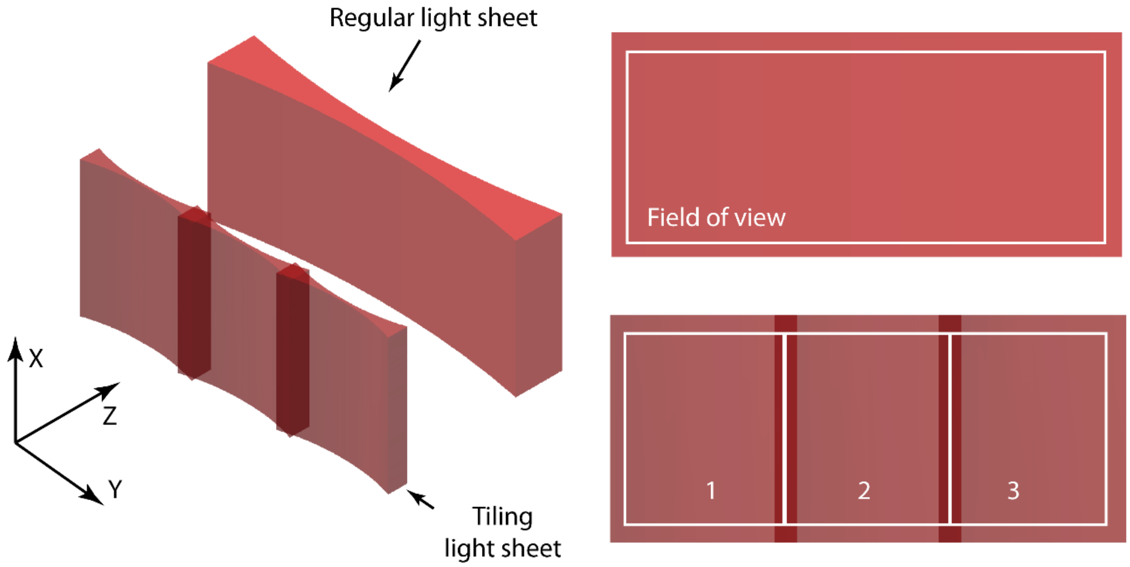

Supplementary Figure 2. The concept of the tiling light sheet selective plane illumination microscopy. The same FOV can be imaged by tiling a smaller but thinner light sheet to obtain higher spatial resolution and better optical sectioning capability compared to a regular light sheet.

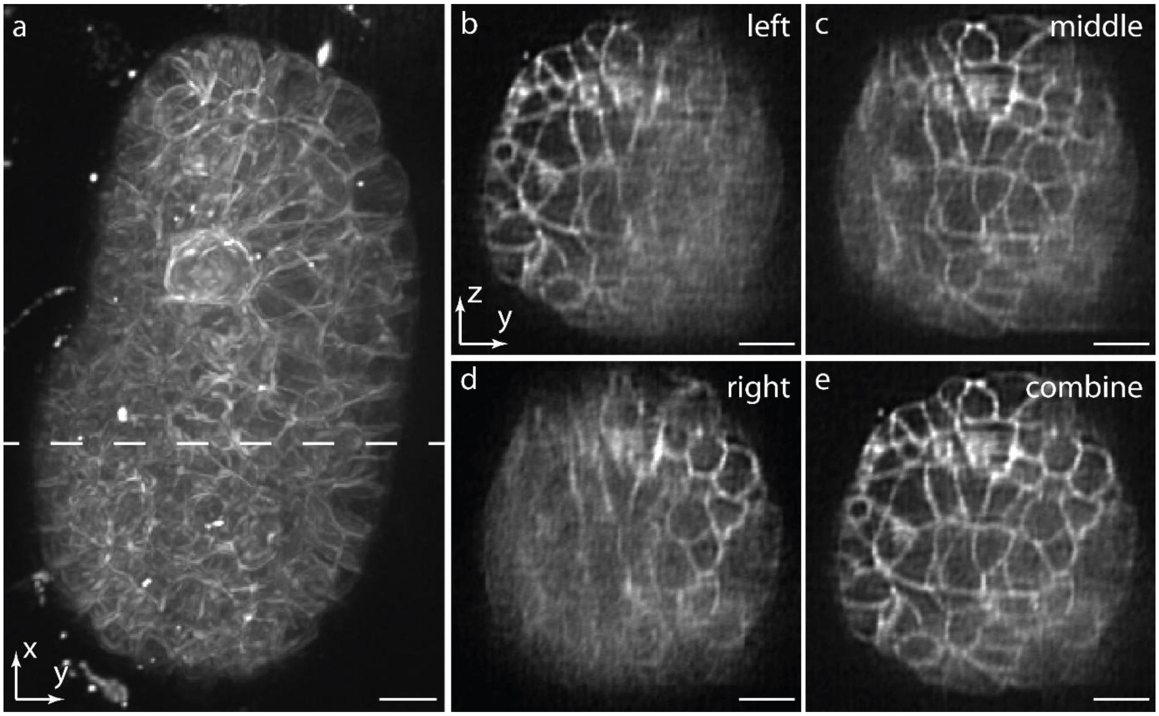

Supplementary Figure 3. The image reconstruction method of TLS-SPIM. Only the fluorescence signal generated at the center of each tiled light sheet is used in the final result. (a) XY maximum intensity projection of a *C. elegans* embryo (OD95, membrane) imaged by TLS-SPIM. (b-d) YZ cross section of the embryo in a through the marked plane, showing the results obtained at three tiling positions. (e) YZ cross section of the reconstructed result. Scale bars, 5 μm.

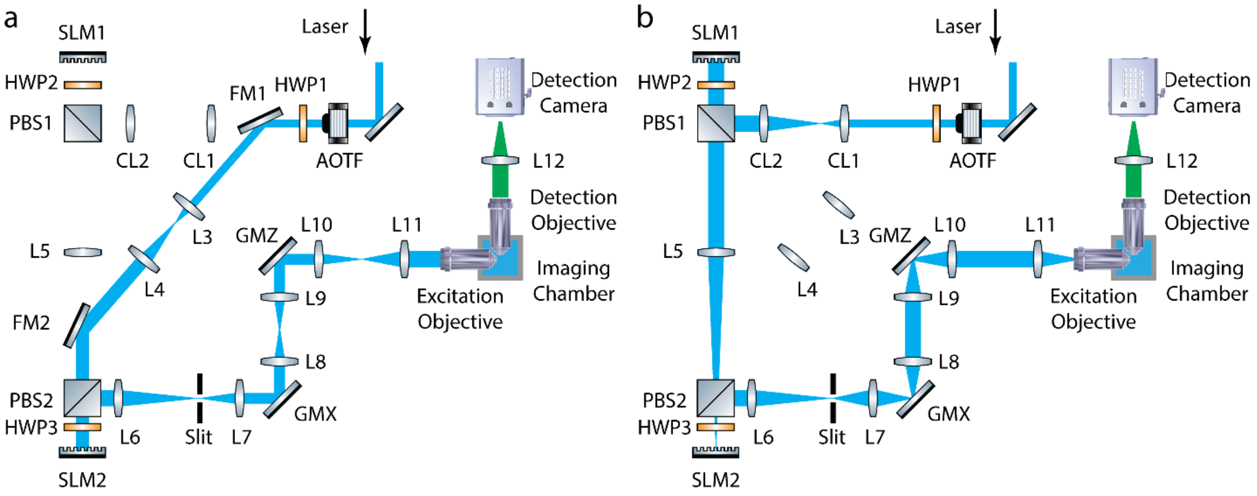

Supplementary Figure 4. Schematic diagram of the tiling light sheet selective plane illumination microscope.

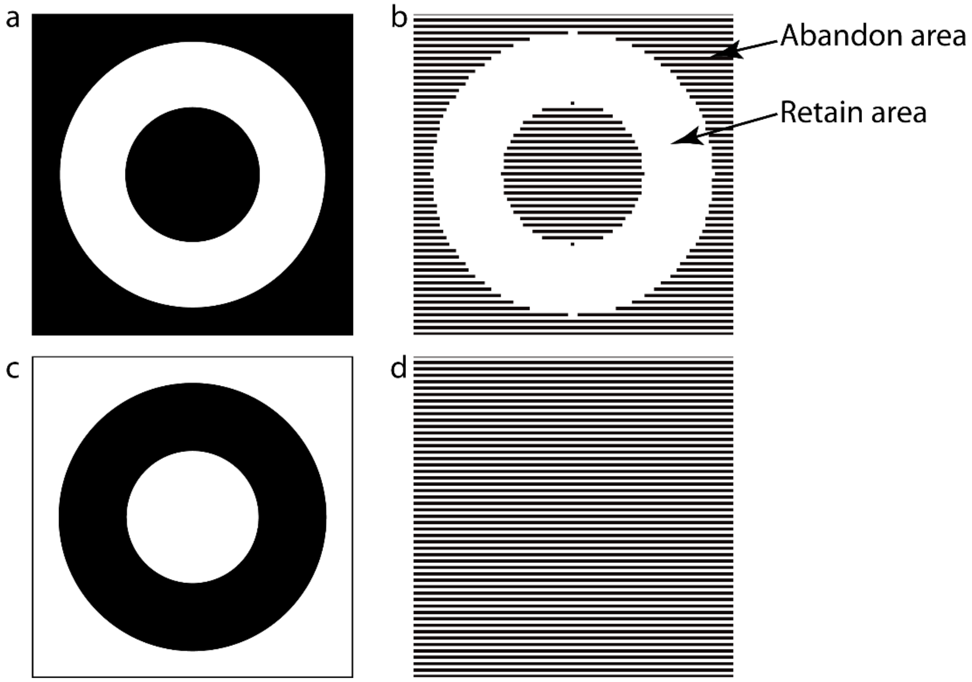

Supplementary Figure 5. Method to adjust the excitation light sheet dimensions in real-time. (a) A circular amplitude mask to create a Bessel beam. (b) The binary phase map applied to SLM2 to replace the amplitude mask in a. It is obtained by multiplying the reverse amplitude mask in c and the high frequency binary phase pattern in d. The light sheet dimension can be adjusted by changing the shape and size of the abandon area and retain area on the SLM2.

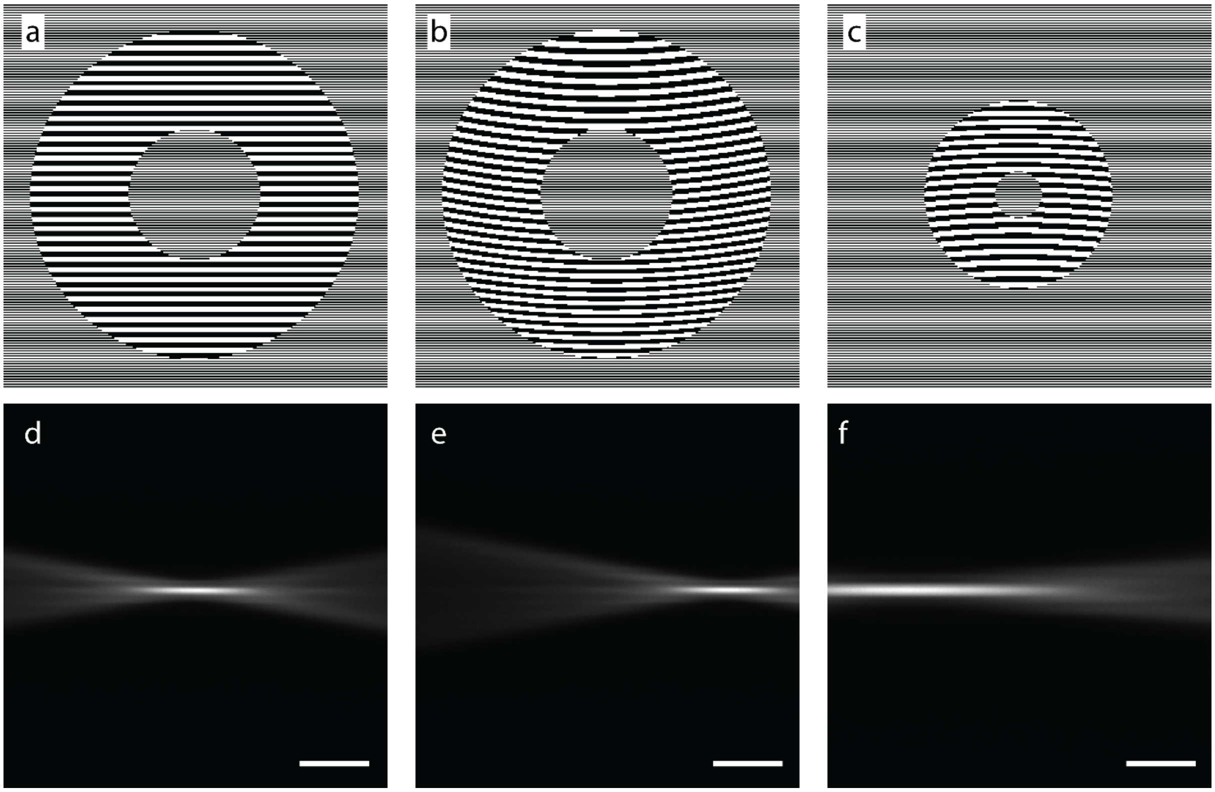

Supplementary Figure 6. Method to tile a single excitation beam. (a-c) The binary phase maps applied to SLM2 to generate and tile a single excitation beam of in d-f. NA_{OD}=0.35, NA_{ID}=0.14 in a and b, NA_{OD}=0.2, NA_{ID}=0.05 in c. Scale bars, 10 μm.

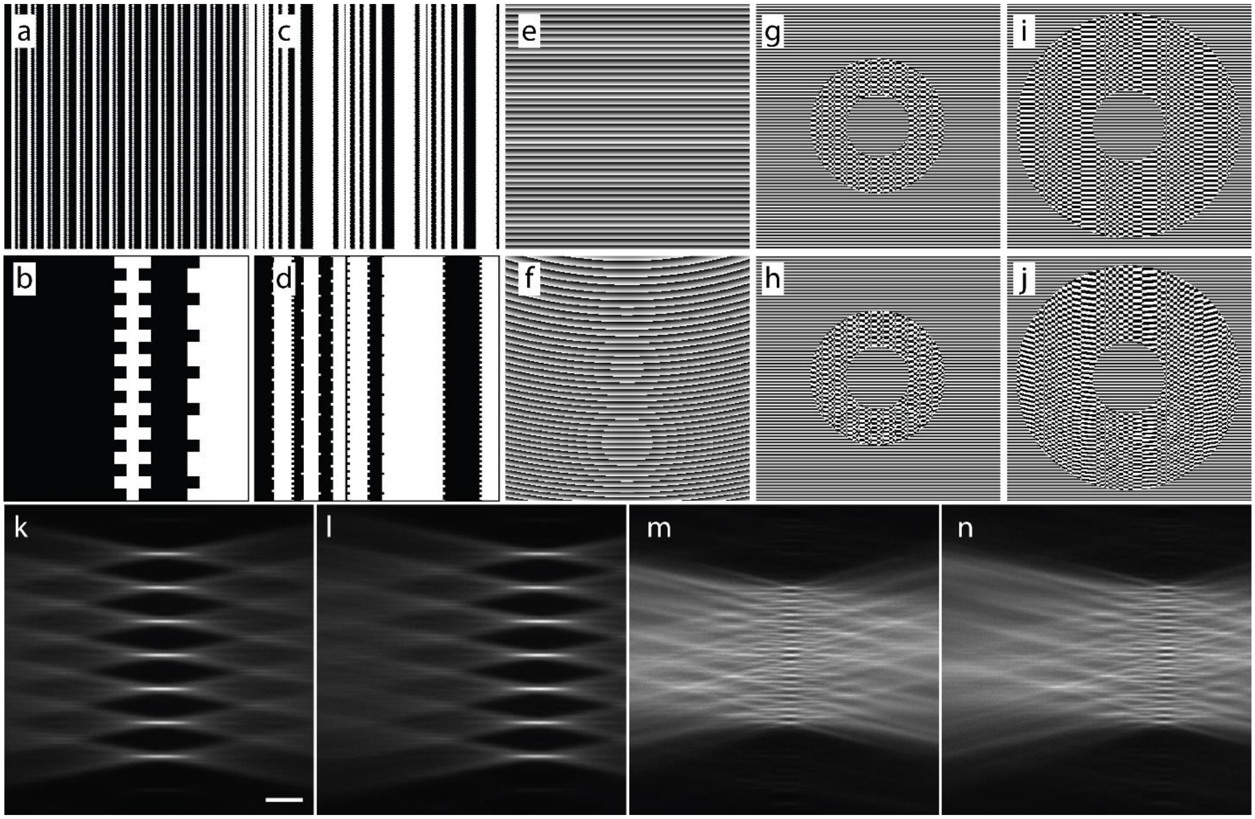

Supplementary Figure 7. Method to generate and tile an incoherent excitation beam array. (a) The Dammann grating pattern used to create a 7 beam array. (b) A full period of the grating in a. (c) the Dammann grating pattern used to create a 21 beam array. (d) A full period of the grating in c. (e, f) The spherical phase maps superimposed to the Dammann gratings to tile the beam array. (g, h) The binary phase maps applied to SLM2 to generate the incoherent beam array in k and l (NA_{OD}=0.35, NA_{ID}=0.14). (i, j) The binary phase maps applied to SLM2 to generate the incoherent beam array in m and n (NA_{OD}=0.5, NA_{ID}=0.14). Scale bar, 10 µm.

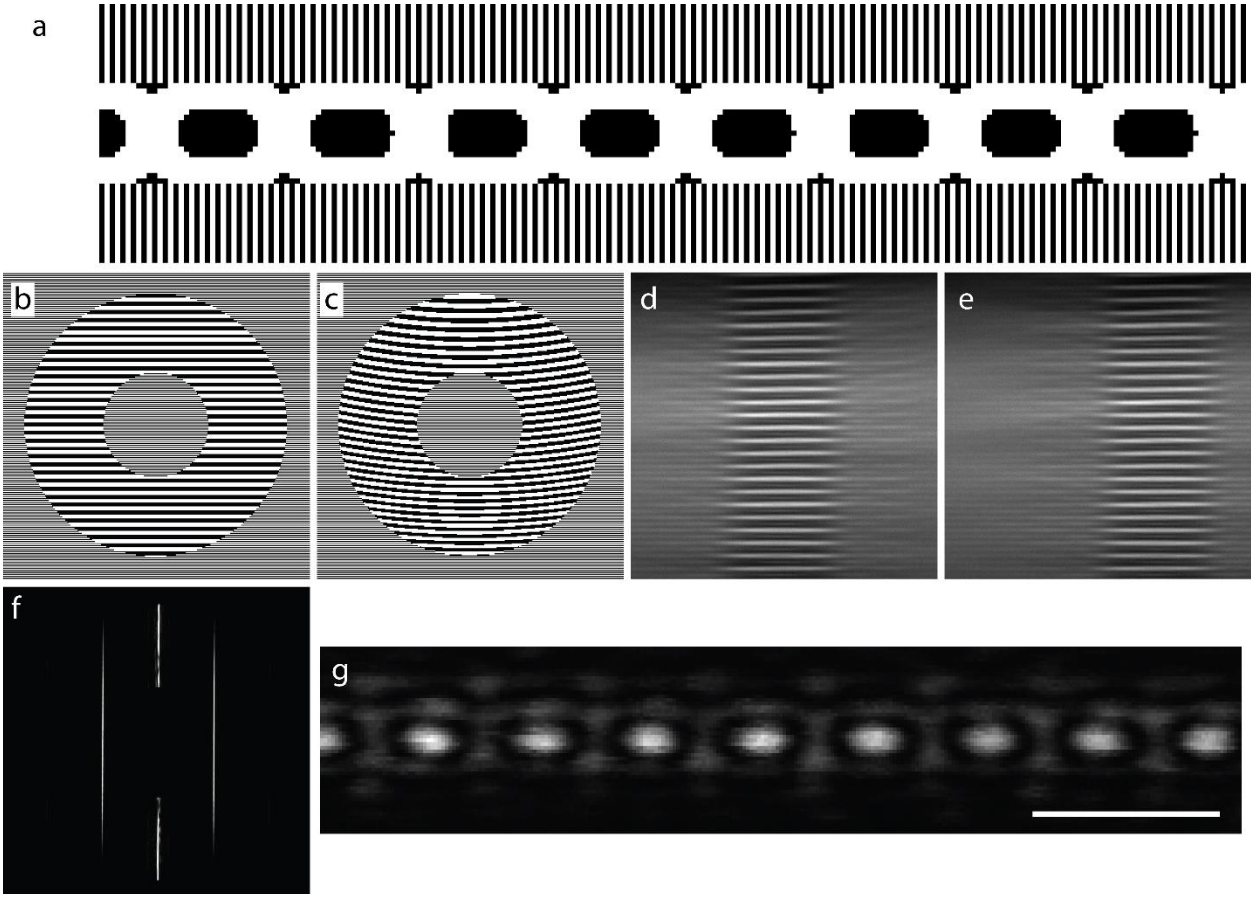

Supplementary Figure 8. Method to generate and tile a coherent Bessel beam array (Lattice light sheet). (a) The binary phase map applied to SLM1 to generate the coherent Bessel beam array in 6e. (b, c) Two phase maps applied to SLM2 (NA_{OD}=0.35, NA_{ID}=0.14) to shape the excitation laser beam and tile the coherent Bessel beam array. (d, e) The coherent Bessel beam array in dye solution, generated by applying 16a, 16b and 16a, 16c to SLM1 and SLM2. (f) Measured laser beam intensity profile at the excitation objective pupil plane. (g) Measured beam array cross section intensity profile at the excitation objective image plane. Scale bar: 5 µm.

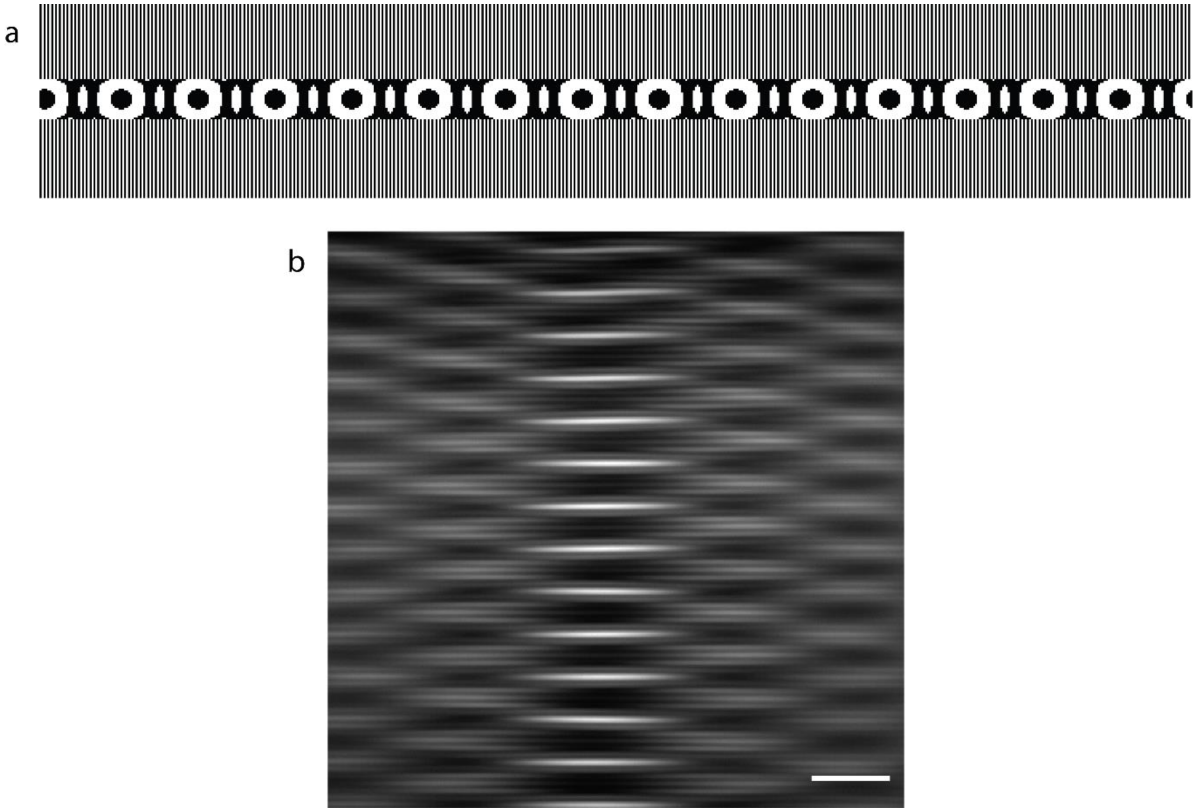

Supplementary Figure 9. Method to generate an incoherent like beam array with two binary SLMs. (a) The binary phase map applied to SLM1 to generate an incoherent like Bessel beam array (NA_{OD}=0.35, NA_{ID}=0.14). (b) The obtained beam array in dye solution by applying the phase map in a to SLM1 and the phase map in 8b to SLM2. Scale bar, 10 μm.

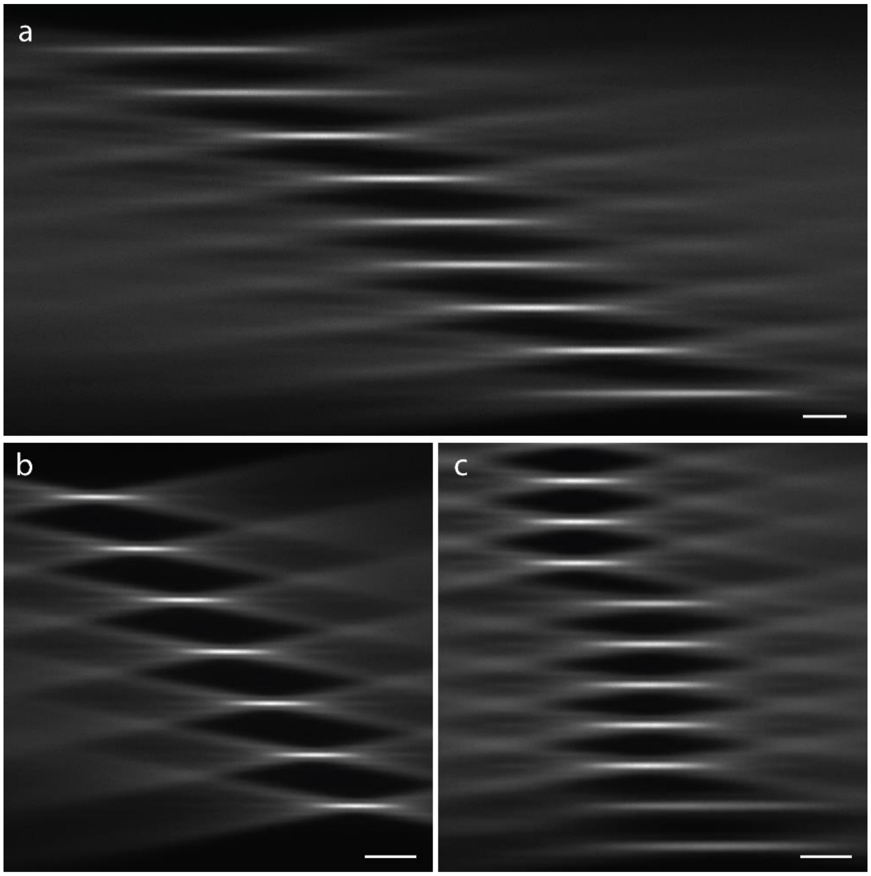

Supplementary Figure 10. Tiling and real-time variation of an excitation beam in TLS-SPIM. (a) An excitation beam (NA_{OD}=0.2, NA_{ID}=0.05) tiled at nine positions during scanning. (b) An excitation beam (NA_{OD}=0.35, NA_{ID}=0.14) tiled at seven positions during scanning. (c) An excitation beam changed in real-time during scanning and tiling.

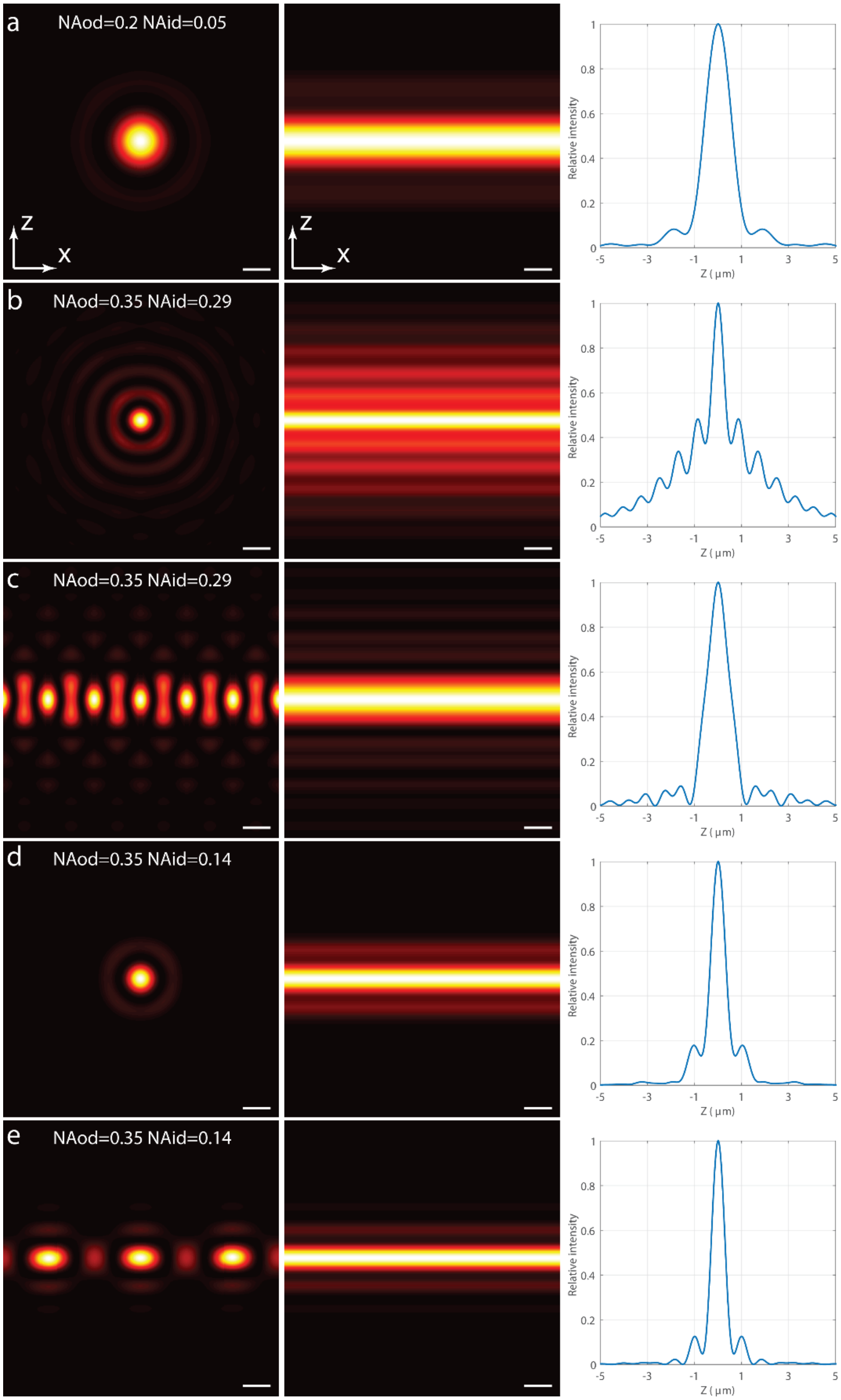

a
NAod=0.2 NAid=0.05
b
NAod=0.35 NAid=0.29
c
NAod=0.35 NAid=0.29
d
NAod=0.35 NAid=0.14
e
NAod=0.35 NAid=0.14
z
x
Relative intensity
Z (μm)

Supplementary Figure 11. Comparison of different excitation light sheets required to image the same FOV of ~33 µm using regular SPIM and TLS-SPIM (three tiling positions). Simulated light sheets are generated by either scanning a Gaussian/Bessel beam (a, b, d) or dithering a coherent Bessel beam array (c, e). (a) NA_{OD}=0.2, NA_{ID}=0.05. (b) NA_{OD}=0.35, NA_{ID}=0.29. (c) NA_{OD}=0.35, NA_{ID}=0.29. (d) NA_{OD}=0.35, NA_{ID}=0.14. (e) NA_{OD}=0.35, NA_{ID}=0.14. Light sheets in b-e provide the same theoretical spatial resolution. Light sheets in d and e give the best optical sectioning capability. Light sheets in a and c have comparable optical sectioning capability. The tiling light sheets used in TLS-SPIM (d, e) are either thinner or have better optical sectioning capability than others. Scale bars, 1 µm.

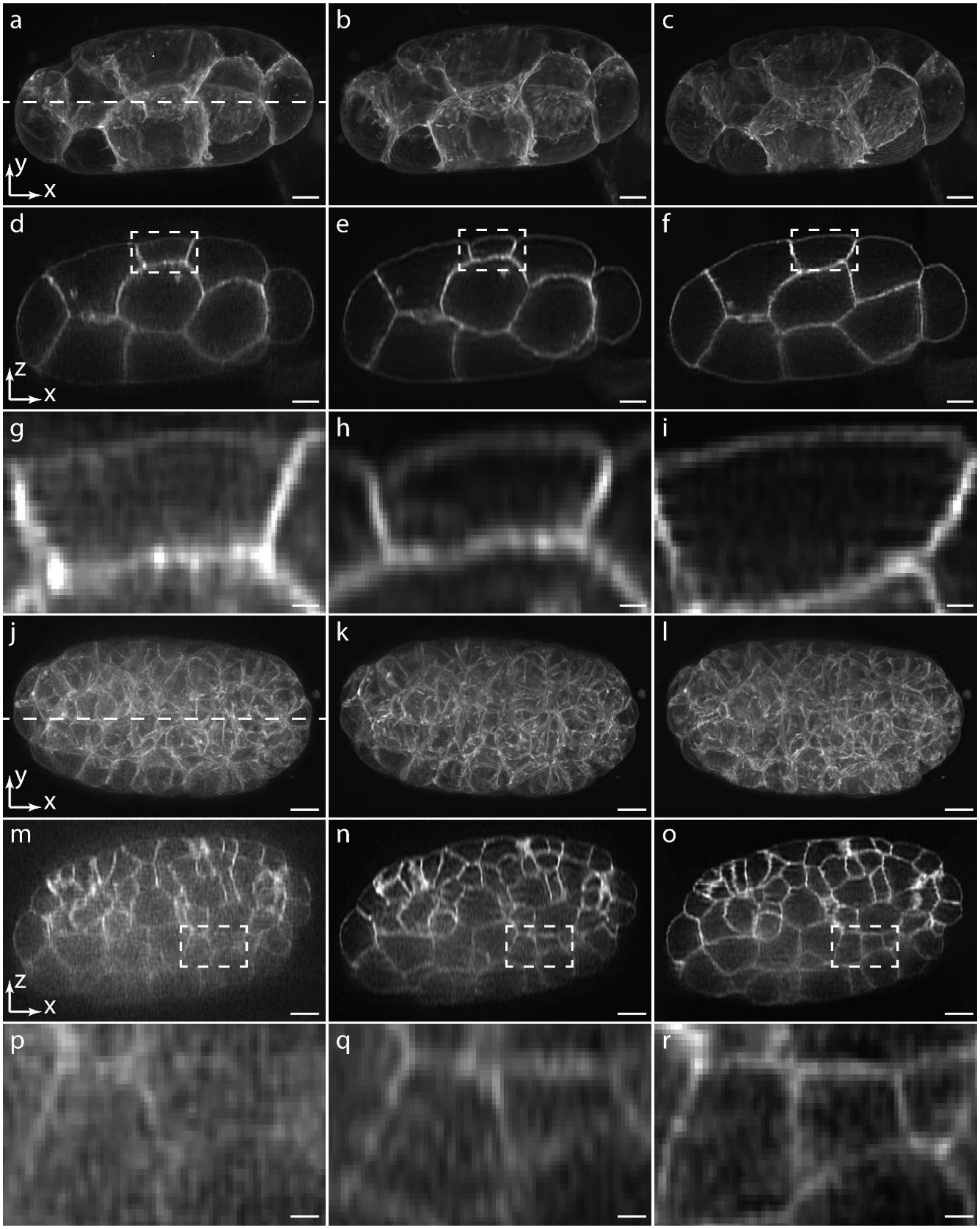

Supplementary Figure 12. 3D imaging ability comparison of regular SPIM (Bessel/Gaussian light sheets) and TLS-SPIM (Bessel light sheet/incoherent seven Bessel beam array, three tiling

positions) on *C. elegans* embryos (OD95, membrane). (a-c) XY maximum intensity projections of a *C. elegans* embryo imaged by regular SPIM with the light sheets in 11a, 11b and TLS-SPIM with the light sheet in 11d. (d-f) YZ axial slices of the embryo in a-c through the marked position. (g-i) Magnified views of the selected areas in d-f. (j-l) XY maximum intensity projections of a later stage *C. elegans* embryo imaged by the three methods. (m-o) YZ axial slices of the embryo through the marked position. (p-r) Magnified views of the selected areas in m-o. The results show that the optical sectioning capability determines if the theoretical spatial resolution can be acquired in practice, especially on complex structures. Both spatial resolution and optical sectioning capability are important to completely resolve a complex structure. Scale bars, 5 µm (a-o), 1 µm (p-r).

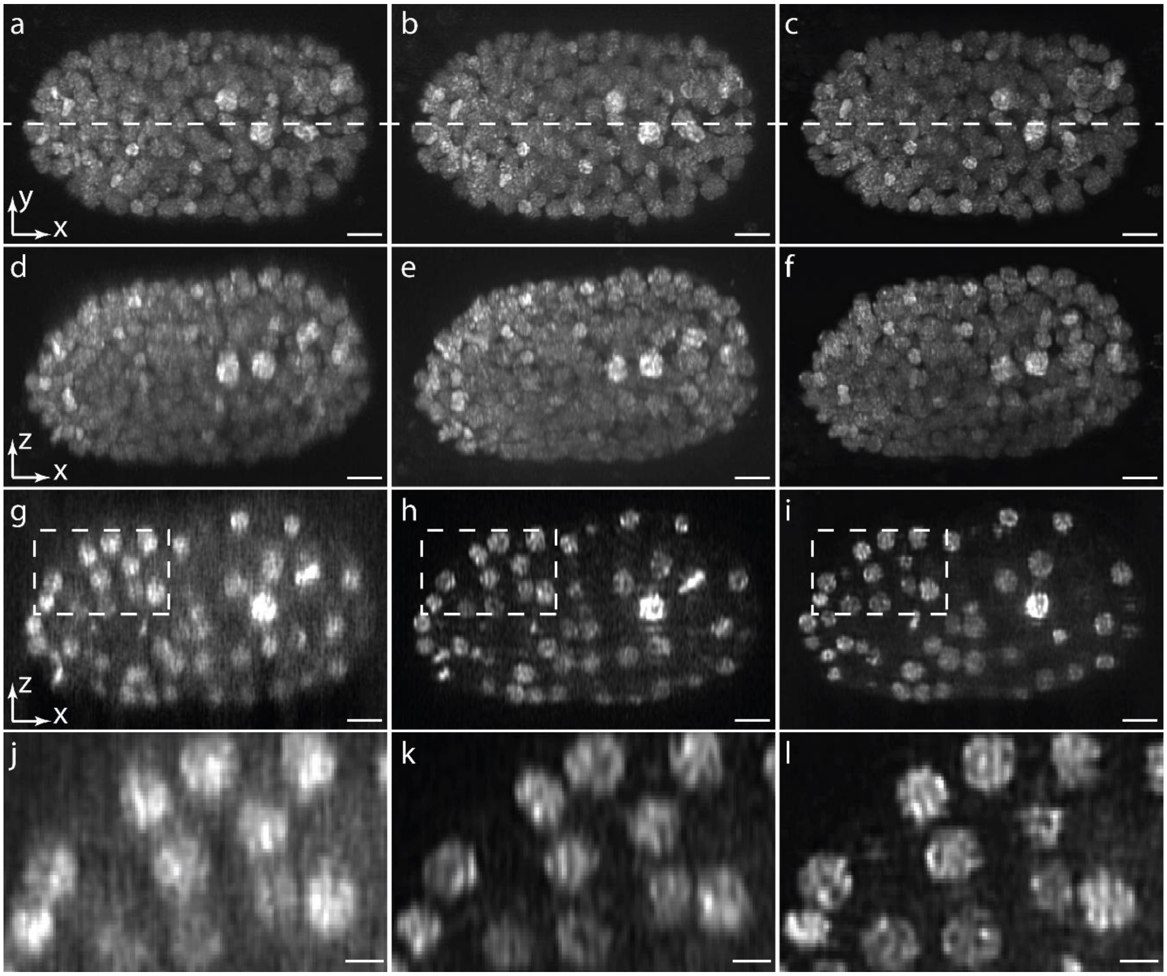

Supplementary Figure 13. 3D imaging ability comparison of regular SPIM (Bessel/Gaussian light sheets) and TLS-SPIM (Bessel light sheet/incoherent seven Bessel beam array, three tiling positions) on a *C. elegans* embryo (RW10029, H2B). (a-c) XY maximum intensity projections of a *C. elegans* embryo imaged by regular SPIM with the light sheets in 11a, 11b and TLS-SPIM with the light sheet in 11d. (d-f) XZ maximum intensity projections of the embryo in a-c. (g-i) YZ axial slices of the embryo in a-c through the marked position. (j-l) Magnified views of the selected areas in g-i. The results show that high spatial resolution is more difficult to be achieved on very condensed structures. The light sheet and imaging mode must be selected carefully based on the desired result. The results in b,e,h,k are good enough to obtain cell nuclei location information, but not subcellular structural information. Scale bar, 5 µm (a-i), 3 µm (j-l).

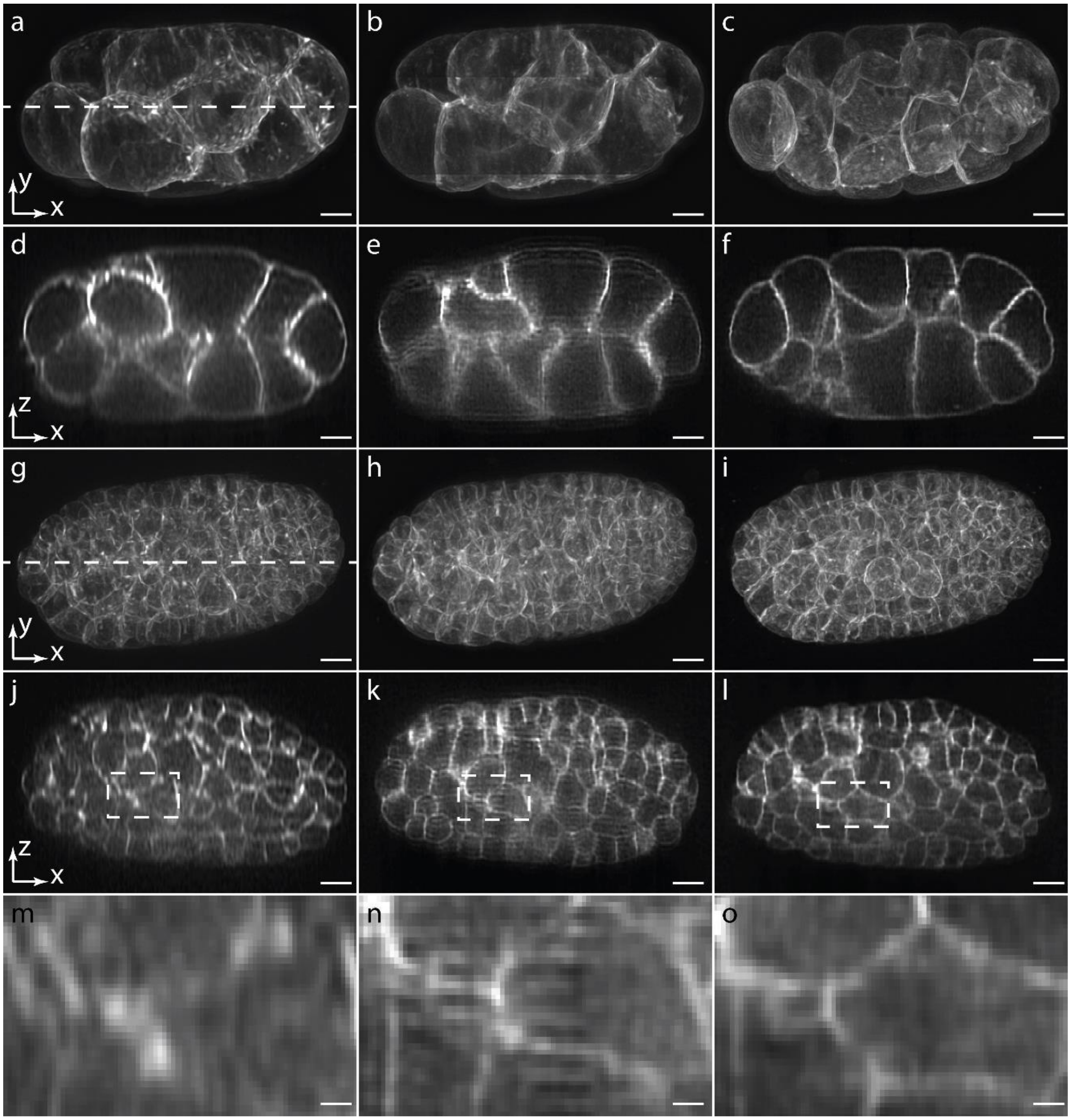

Supplementary Figure 14. 3D imaging ability comparison of Lattice light sheet microscopy and TLS-SPIM (Lattice light sheet/coherent Bessel beam array and Bessel light sheet/incoherent seven Bessel beam array, three tiling positions) on *C. elegans* embryos (OD95, membrane). (a-c) XY maximum intensity projections of a *C. elegans* embryo imaged by regular Lattice light sheet microscopy with the light sheet in 11c and TLS-SPIM with the Lattice light sheet in 11e and the Bessel light sheet in 11d. (d-f) YZ axial slices of the embryo in a-c through marked position. (g-

i) XY maximum intensity projection of a later stage *C. elegans* embryo imaged by the three methods. (j-l) YZ axial slices of the embryo in g-i through the marked position. (m-o) Magnified views of the selected areas in j-l. The results show TLS-SPIM with a Bessel light sheet has better 3D imaging ability than regular Lattice light sheet microscopy, while Lattice light sheets are more sensitive to sample aberration as we observed less aberration effect on late stage *C. elegans* embryos (j, k) and fluorescent particles embed in agarose gel (Supplementary Fig. 15). Scale bars, 5 µm (a-l), 1 µm (m-o).

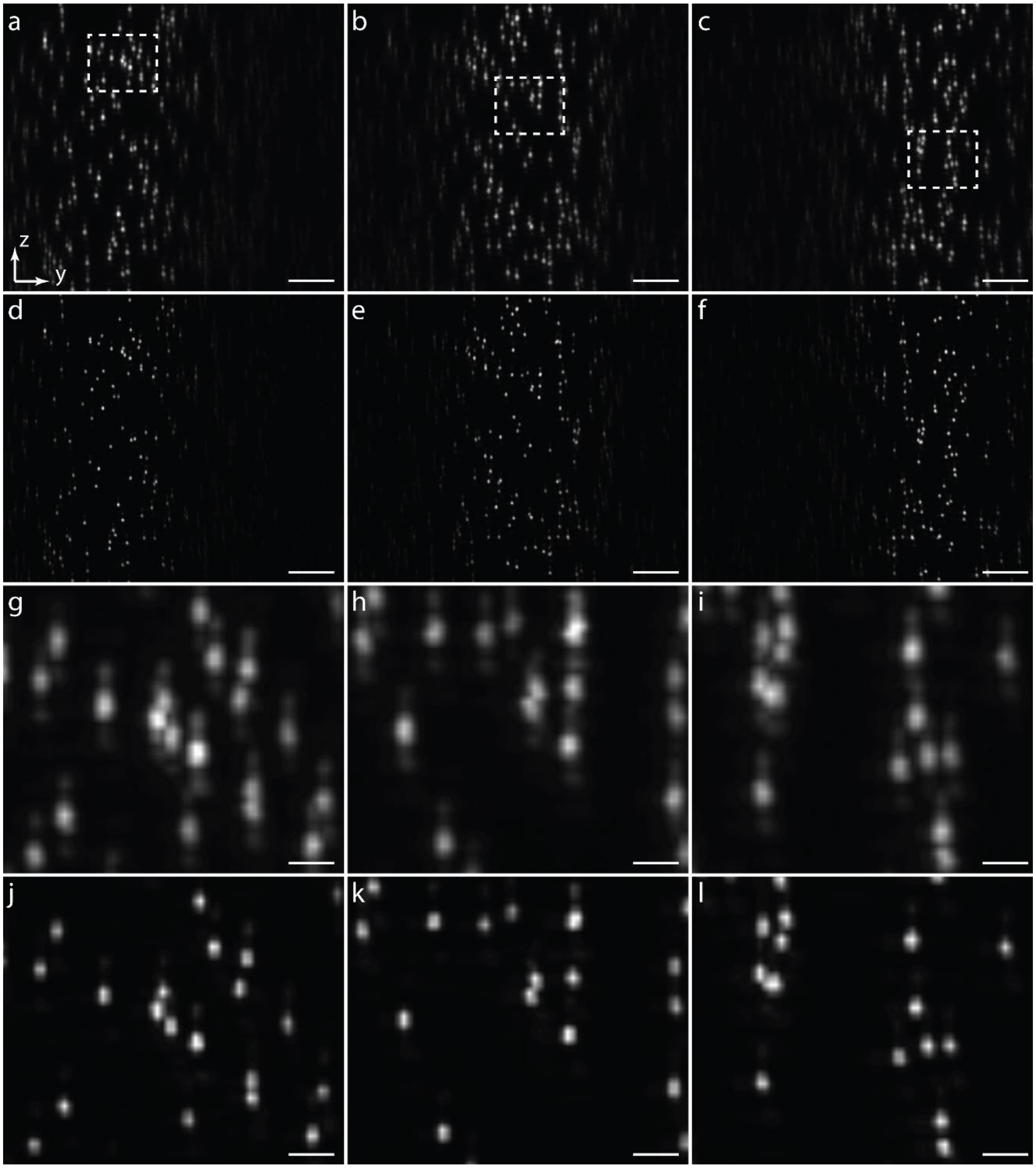

Supplementary Figure 15. Fluorescent particles embedded in agarose gel imaged by TLS-SPIM with a tiling Lattice light sheet in 11e. (a-c) YZ maximum intensity projections of the same FOV imaged by tiling the lattice light sheet at the left, middle and right of the FOV. (d-f) YZ maximum intensity projections of the deconvolved results of a-c. (g-i) Magnified views of the

selected areas in a-c. (j-l) Magnified views of the selected areas in d-f. Scale bars, 5 μm (a-f), 1 μm (g-l).

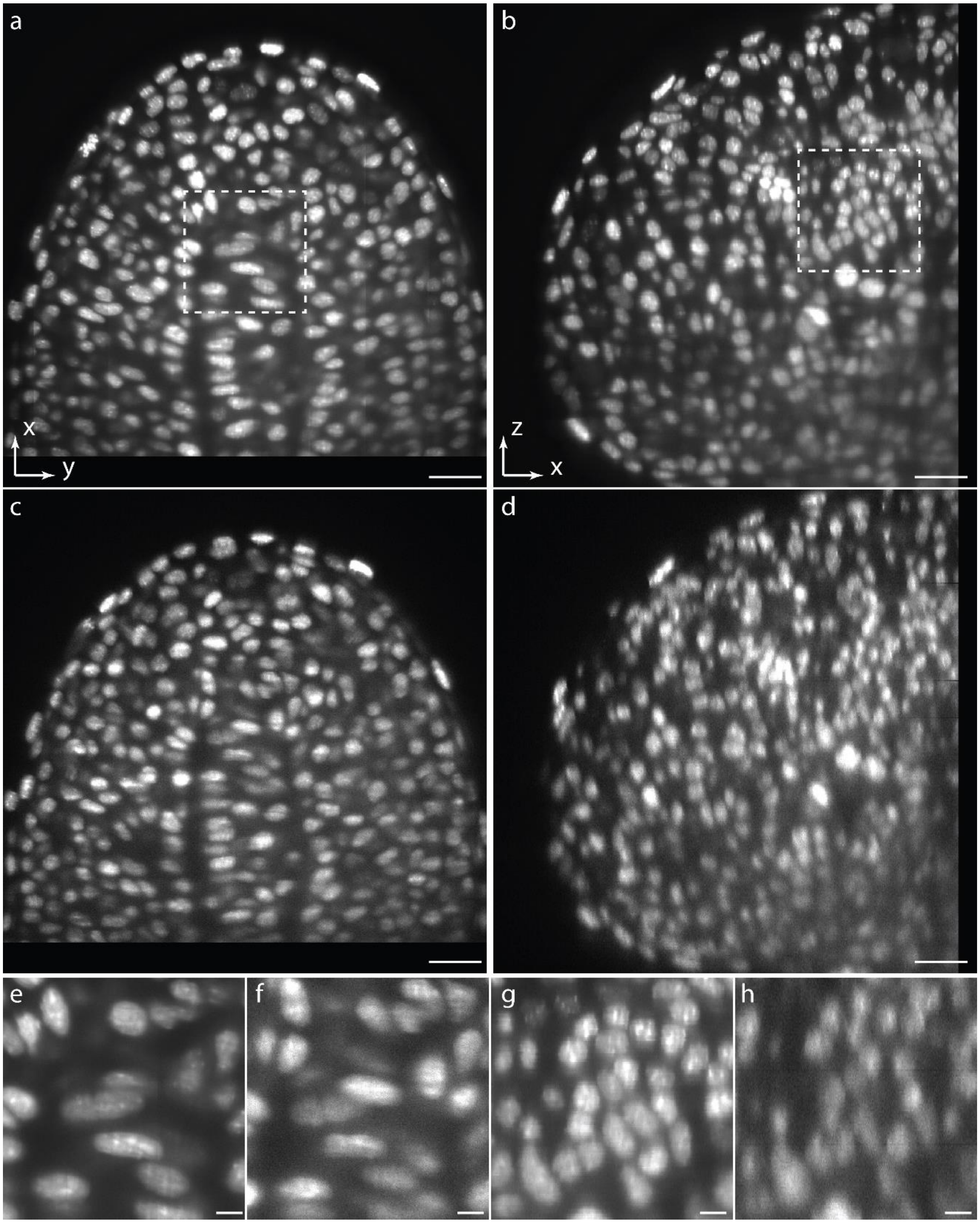

Supplementary Figure 16. 3D imaging ability comparison of regular SPIM and TLS-SPIM on a nucleus-labeled zebrafish embryo. (a, b) XY and XZ orthogonal slices of a ~15 hpf zebrafish embryo tailbud imaged by TLS-SPIM (NA_{OD}=0.2, NA_{ID}=0.05, single excitation beam, nine

tiling positions, raw image). (c, d) XY and XZ orthogonal slices of the same embryo imaged by regular SPIM with a Gaussian light sheet (NA_{OD}=0.07, single excitation beam, raw image). (e, f) Magnified views of the selected areas in a and c. (g, h) Magnified views of the selected area in b and d. Scale bars, 20 µm (a-d), 5 µm (e-h).

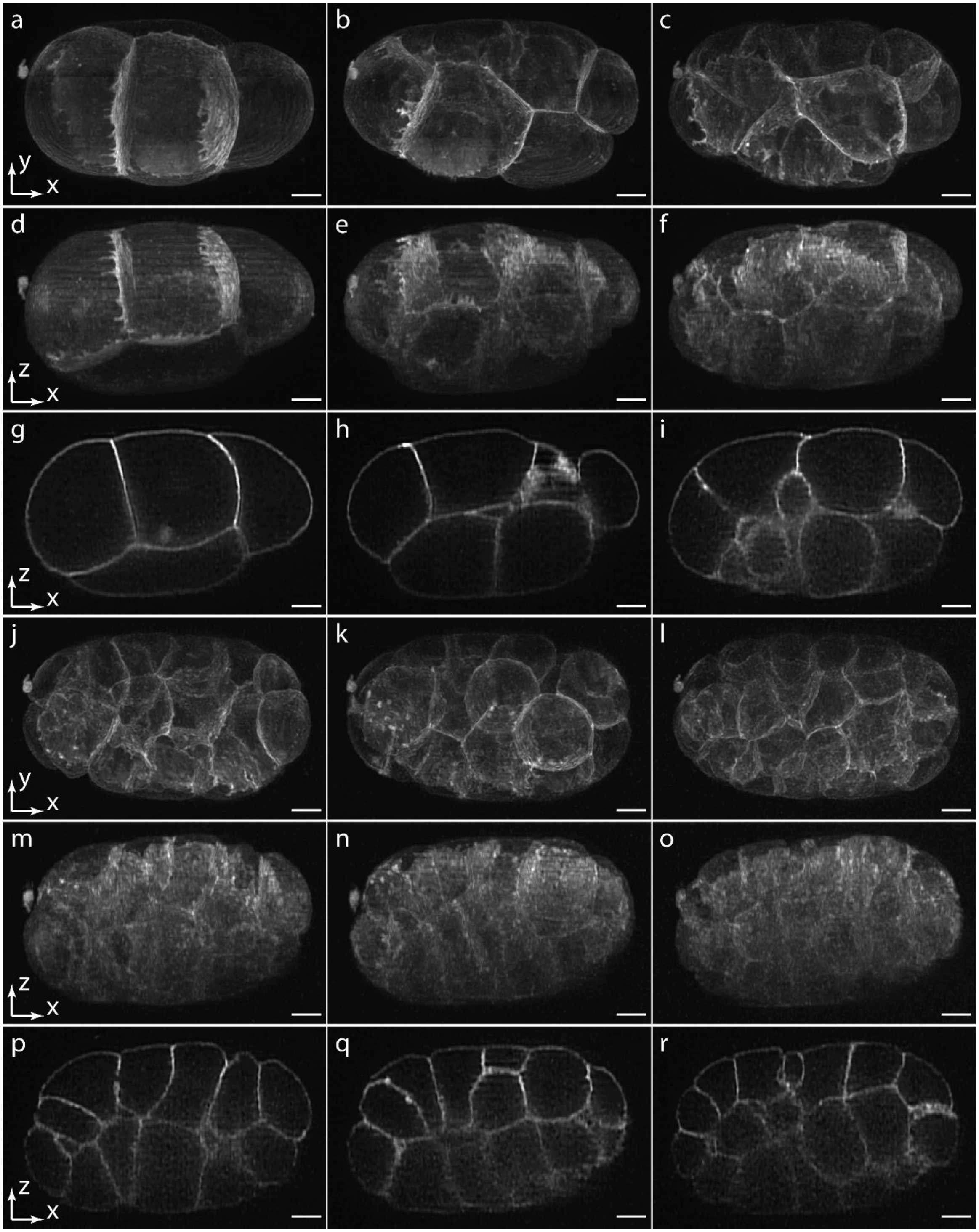

Supplementary figure 17: Selected time points of a *C. elegans* embryo (OD95) imaged by TLS-SPIM for 167 time points (NA_{OD}=0.35, NA_{ID}=0.14, Bessel light sheet/incoherent seven Bessel

beam array, three tiling positions). (a-c, j-l) Lateral maximal intensity projections of the embryo at time points of 25, 50, 75, 100, 125 and 150. (d-f, m-o) Axial maximal intensity projections of the embryo at these time points. (g-i, p-r) XZ axial slices the embryo through the longitudinal axial at these time points. The result show that both high spatial resolution and good SNR are maintained through the whole imaging process. Scale bars, 5 μm.
